# Comparison of the Hi-C, GAM and SPRITE methods using polymer models of chromatin

**DOI:** 10.1038/s41592-021-01135-1

**Published:** 2021-05-07

**Authors:** Luca Fiorillo, Francesco Musella, Mattia Conte, Rieke Kempfer, Andrea M. Chiariello, Simona Bianco, Alexander Kukalev, Ibai Irastorza-Azcarate, Andrea Esposito, Alex Abraham, Antonella Prisco, Ana Pombo, Mario Nicodemi

**Affiliations:** 1 Dipartimento di Fisica, Università di Napoli Federico II and INFN Napoli, Complesso Universitario di Monte Sant’Angelo, Naples, Italy; 2 Berlin Institute for Medical Systems Biology, Max-Delbrück Centre for Molecular Medicine, Berlin, Germany; 3 Humboldt-Universität zu Berlin, Berlin, Germany; 4 CNR-IGB, Naples, Italy; 5 Berlin Institute of Health, Berlin, Germany

**Keywords:** Computational models, Computational biophysics, Biological techniques

## Abstract

Hi-C, split-pool recognition of interactions by tag extension (SPRITE) and genome architecture mapping (GAM) are powerful technologies utilized to probe chromatin interactions genome wide, but how faithfully they capture three-dimensional (3D) contacts and how they perform relative to each other is unclear, as no benchmark exists. Here, we compare these methods in silico in a simplified, yet controlled, framework against known 3D structures of polymer models of murine and human loci, which can recapitulate Hi-C, GAM and SPRITE experiments and multiplexed fluorescence in situ hybridization (FISH) single-molecule conformations. We find that in silico Hi-C, GAM and SPRITE bulk data are faithful to the reference 3D structures whereas single-cell data reflect strong variability among single molecules. The minimal number of cells required in replicate experiments to return statistically similar contacts is different across the technologies, being lowest in SPRITE and highest in GAM under the same conditions. Noise-to-signal levels follow an inverse power law with detection efficiency and grow with genomic distance differently among the three methods, being lowest in GAM for genomic separations >1 Mb.

## Abstract (web summary)

This Analysis reports a computational approach to implement Hi-C, SPRITE and GAM, which allows researchers to assess the performances of the three technologies to capture DNA contacts in chromatin three-dimensional models.

## Main

To access genome-wide chromosome physical interactions in the cell nucleus, high-throughput technologies have been developed^1^ that exploit the power of sequencing, spanning from chromosome conformation capture (3C)-based methods, such as Hi-C and its derivatives^2–8^, to GAM^9^ and SPRITE^10^. They have shown that the mammalian genome has a complex 3D organization crucial in the regulation of genomic functions^11–17^, which encompasses DNA loops^4^, megabase-sized topologically-associated domains (TADs)^18,19^ and higher-order structures such as metaTADs^20^ and A/B compartments^2^. However, while microscopy is rapidly advancing^21–24^, it remains unclear to what extent those technologies are faithful to the underlying 3D structure of the genome and how they perform relative to each other in different applications, because they return distinct measures of interactions and no benchmark exists. Is GAM as faithful to genome architecture as SPRITE? Are single-cell Hi-C data reliable or dominated by noise? How many cells are required to attain statistically significant results? How does detection efficiency impact experimental outcomes? Which method is more suited to capture interactions at large genomic distances? Here we answer those questions by comparison of the in silico performance of Hi-C, GAM and SPRITE to capture the architecture of a known set of polymer 3D structures from validated models of real chromosomal loci.

Hi-C methods have revolutionized the field of chromosome architecture and are widely used. They provide a measure of the abundance of pairwise interactions—that is, a Hi-C contact frequency map—by sequencing the ligation products of DNA fragments that are in close spatial proximity in the nucleus^2,4^. GAM probes the 3D proximity of DNA sites by sequencing the genomic content of thin, cryosectioned and laser-microdissected slices from the nuclei of cells fixed under optimal preservation conditions^1,9^: physically distant DNA sites are unlikely to cosegregate in the same thin slice whereas physically proximal sites do so. The output of a GAM experiment is a cosegregation map—that is, the frequency with which pairs (or groups) of genomic regions are found in the same slice, as detected by sequencing. Nonrandom DNA interaction probabilities in single cells can then be reconstructed by the use of statistical tools such as statistical inference of cosegregation (SLICE)^9^. Finally, SPRITE^10^ relies on the sequencing of barcoded DNA: after DNA crosslinking and fragmentation in isolated nuclei (as in Hi-C), interacting chromatin complexes are uniquely barcoded via a split-pool method and identified by sequencing. SPRITE interaction maps can be extracted from DNA segments with the same barcode, which must originate from the same interacting complex.

To compare Hi-C, GAM and SPRITE, we ran a computational experiment implementing all three methods in silico on an ensemble of known 3D polymer structures, and analyzed their outputs in such a simplified, yet fully controlled, framework. To facilitate comparison with real experimental data, rather than using arbitrary polymer conformations, we focused on the models of three 6-Mb genomic regions around the *Sox9* and *HoxD* genes in mouse embryonic stem cells (mESC)^25,26^ and around the *Epha4* gene in mouse CHLX-12 cells^27^, and of a 2.5-Mb locus in human HCT116 cells^28^. These loci are particularly interesting because disease-linked structural variants around the *Sox9* and *Epha4* genes have been shown to induce gene misexpression as a consequence of the rewiring of contacts with local enhancers^16,27,29^, and the *HoxD* locus has a specific 3D compartmentalization thought to control transcriptional states during differentiation^30,31^.

Different computational approaches^32–36^ and polymer models^25,27,37–47^ have been discussed to reconstruct chromatin 3D conformations. Here, we focus on the String&Binders (SBS) model^27,38,47^ that was shown to reproduce accurately the architecture of chromosomal loci^25–28^. The SBS model of each of the loci considered was inferred from available Hi-C data and used to derive an ensemble of 3D structures. Those 3D structures were in turn employed to benchmark the performances of in silico Hi-C, SPRITE and GAM experiments. For the *Sox9* locus, we also analyzed a polymer model inferred from GAM data^48^ and found similar results.

To validate our approach, we demonstrated that in silico average Hi-C, GAM and SPRITE data all successfully compare against corresponding independent experiments, and that our model returns a bona fide representation of chromatin conformations by comparison against independent, single-cell, multiplexed FISH imaging data available for the human HCT116 cell locus^22^. That provides evidence that the architecture of the loci considered is well described by our polymer models and that they can be used to compare the performance of the three technologies with respect to key experimental parameters, including detection efficiency, genomic separation and cell numbers.

We found that in silico Hi-C, GAM and SPRITE bulk data are overall faithful to the reference 3D structures of the polymer models of the loci considered. The intrinsic variability of single-molecule conformations renders single-cell contact data much less faithful to the underlying 3D structure and strongly different across replicates. We identified the minimal number of cells required for replicate experiments to return statistically consistent data, which is shown to be different across the technologies—lowest in SPRITE and highest in GAM under the same conditions. The noise-to-signal level in contact matrices grows as a power law by decreasing efficiency, which implies that experiments using large cell numbers may be required to reduce noise effects, and it varies with genomic distance differently in the three methods, with GAM being the least affected by noise at larger genomic separations.

## Results

### Derivation of in silico contact maps from known single-molecule 3D structures

**Fig. 1:**
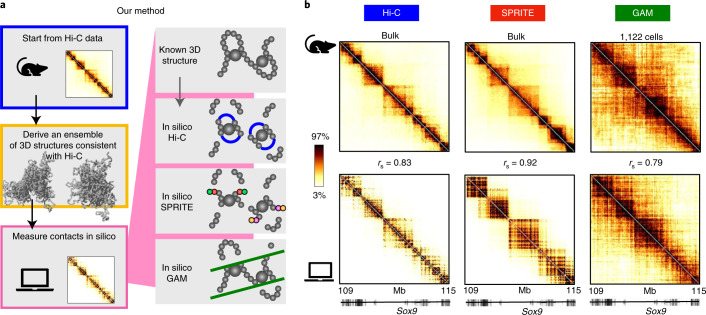
In silico Hi-C, SPRITE and GAM average contact maps match experimental data. **a**, Illustration of computational experiments. An ensemble of single-molecule 3D structures of the polymer model of the DNA locus of interest is derived from bulk Hi-C data using the the PRISMR procedure^27^ and polymer physics simulations. **b**, The model 3D conformations, inferred from Hi-C data only, return average contact maps (bottom) that can be compared to Hi-C and independent SPRITE and GAM experimental data (top) in the study of the *Sox9* locus (chr11:109–115 Mb, mm9) in mESC. Experimental Hi-C and SPRITE maps are derived from bulk data^10,19^, while GAM data are from a new dataset constructed from 1,122 F123 cells (Methods) and, correspondingly, the in silico maps. The color scale represents the percentiles of each dataset. Values of Spearman correlation *r*_s_ between model and experiment are reported. Pearson and HiCRep correlations are reported in Supplementary Table 1a.

For comparison of in silico Hi-C, GAM and SPRITE data, we focused first on the case study of a 6-Mb region around the *Sox9* gene (chr11:109–115 Mb, mm9) in mESCs and its SBS polymer model^25^. The SBS is a model of chromatin where molecules, such as transcription factors, form DNA loops by bridging distal cognate binding sites^47^. It has been shown to accurately describe Hi-C, GAM and FISH data across loci and cell types^20,25–27,38,48–50^. The genomic locations of the binding sites of the *Sox9* locus model were inferred from its Hi-C data^19^ by the PRISMR algorithm^25,27^, a machine learning method that determines the minimal set of binding sites (and cognate binders) best describing the input data from only polymer physics (Methods). Here, we considered the published model of the locus at 40-kb resolution and explored an ensemble of single-molecule 3D polymer structures derived by molecular dynamics (MD) simulations in the thermodynamic steady state of the system (Fig. 1a and Methods).

To derive Hi-C, GAM and SPRITE in silico contact data, we computationally implemented the steps of the three methods on those 3D structures (Fig. 1a). In brief, with in silico Hi-C we fragmented in equal segments the two polymer chains representing the two *Sox9* alleles in each cell, ligated crosslinked fragments and counted ligation products to derive an in silico analog of Hi-C contact frequencies (Methods). The overall efficiency of the process is the product of the in silico crosslinking, digestion, biotinylation, ligation and sequencing efficiencies. In silico SPRITE was similarly implemented by counting chain fragments tagged with the same barcode. Finally, in silico GAM was performed by cutting randomly oriented slices from a sphere (representing the nucleus) where two single-molecule 3D structures (the two ‘alleles') had been randomly positioned, and by listing the polymer sites falling within each slice, to derive the cosegregation matrix (Methods). The overall efficiency comprises the detection and sequencing efficiency of such sites. The nuclear radius and slice thickness are set to match typical experimental values^9,25^ (Methods).

By such a procedure we derived in silico contact maps of the known polymer 3D structures and investigated how the output of the different technologies is affected by the overall detection efficiency, by the number of pairs, *N*, of 3D single-molecule structures included in the analysis (below, for simplicity, we refer to *N* as the number of in silico cells) and how that varies with genomic separation.

### In silico Hi-C, SPRITE and GAM reproduce experimental 3D structure data

Because our polymer model is inferred from Hi-C data^19^, to check that the derived in silico bulk Hi-C map—that is, contact data averaged over the ensemble of 3D structures—reproduces real bulk Hi-C data^19^ in the *Sox9* locus (Fig. 1b), we measured their correlation and found that the coefficients Spearman (*r*_s_), Pearson (*r*) and HiCRep (stratum adjusted correlation coefficient (scc))^51^ have high values: *r*_s_ = 0.83, *r* = 0.83 and scc = 0.80, respectively (Supplementary Table 1a), as previously reported^25^. Similar results were obtained for the *HoxD* locus in mESC and the *Epha4* locus in CHLX-12 cells (Supplementary Figs. 1a,b and 2a and Methods).

To validate our approach, we next compared the in silico SPRITE and GAM contact matrices derived from the same ensemble of model 3D structures with the corresponding, independent SPRITE and GAM experimental matrices, and we found high correlations between model and experiment—respectively, *r*_s_ = 0.92 and *r*_s_ = 0.79, *r* = 0.75 and *r* = 0.80 (Fig. 1b and Supplementary Table 1a). The HiCRep score, albeit designed for comparison of Hi-C data, is also statistically significantly high—respectively, scc = 0.57 and scc = 0.40, (Methods and Supplementary Fig. 3). In the comparison we used published SPRITE bulk mESC data^10^ and a GAM dataset produced for the 4D Nucleome Consortium^52^ (Methods) composed of 1,122 nuclear slices from F123 mES cells, compared with the output from 1,122 in silico slices. The lower correlation between experimental and in silico GAM contact matrices (derived from Hi-C-based polymers) raises the possibility that Hi-C and GAM may capture some distinct specific contacts, although those differences could derive from noise. Again, similar results were found for the mESC *HoxD* locus (Supplementary Fig. 1a,b; SPRITE and GAM data are not available for the *Epha4* locus in CHLX-12 cells).

**Fig. 2:**
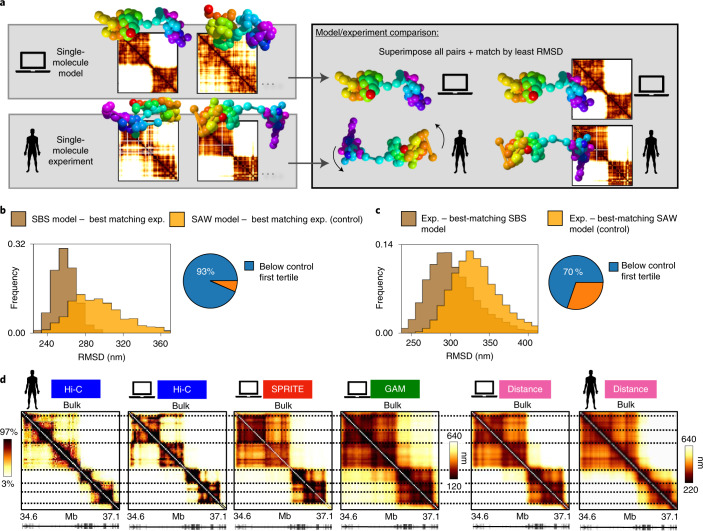
Model 3D structures are a bona fide representation of chromatin conformations in single cells. **a**, Illustration of the method employed to associate single-molecule model 3D structures (top) with a corresponding chromatin 3D structure from imaging data (bottom) via RMSD^28,54^ (Main Text and Methods). **b**, In the 2.5-Mb human HCT116 locus considered, where imaged single-cell chromatin conformations are available^22^, the distribution of RMSD between the 3D structures of the SBS model and their best-matching experimental structure (brown), and that of RMSD between SAW structures (control) and their best-matching experimental conformation (orange), are shown. The two distributions are statistically different (two-sided Mann–Whitney *U*-test *P* = 0); the pie chart highlights that 93% of the former is below the first tertile of the latter. **c**, Distributions of RMSD between the experimental (exp.) structures and best-matching conformation from the SBS model (brown) and the SAW model (orange) are statistically different (*P* = 0), and 70% of the former is below the first tertile of the latter. **d**, In the HCT116 locus, experimental and in silico Hi-C contact matrices (two heatmaps on the left), in silico SPRITE and GAM matrices (two heatmaps in the middle) and model and experimental average distance matrices (two heatmaps on the right) are shown.

To demonstrate that the SBS model 3D structures are a bona fide representation of chromatin conformations in single cells, we took advantage of published multiplex FISH super-resolution microscopy data^22^ for a 2.5-Mb region in human HCT116 cells (chr21:34.6–37.1 Mb), because we can compare experimental and model single-molecule 3D structures^22,28^ and Hi-C data^53^ (GAM and SPRITE data are not available for that cell type). We repeated the procedure described for the *Sox9* locus and compared all-against-all the model-predicted 3D structures with those from imaging data^22^ (Fig. 2a). To find the best match between model and experimental structures, each SBS model single-molecule conformation was univocally associated with a corresponding imaged 3D structure, by searching the minimum root mean squared deviation^28,54^ (RMSD) of the coordinates of pairs of rotated and centered 3D structures. To test the significance of the association, as a control we considered self-avoiding random-walk (SAW) polymer chains having the same number of beads and the same average gyration radius—that is, same size—as the real images of the locus (Methods), and we univocally associated each SAW structure with an experimental structure by the least RMSD criterion. Next, we compared the RMSD distribution between SBS structures and their best-matching experimental structure to that between SAW structures and their best-matching experiment (Fig. 2b). The two distributions were found to be statistically different (two-sided Mann–Whitney *U*-test *P* = 0), with 93% of the former falling below the first tertile of the latter (Fig. 2b). We also compared the RMSD distribution between the experimental structures and their best-matching SBS model conformation with the control distribution of RMSD between experimental structures and their best-matching SAW conformation (Fig. 2c). Again, the two distributions are statistically different (*P* = 0), with 70% of the former below the first tertile of the control. Finally, we verified that the distribution of RMSD between experimental structures and their best-matching SBS model conformation is statistically indistinguishable from that between the best-matching pairs of experimental structures (*P* = 0.15).

To further validate the model, we checked that the SBS-predicted and microscopy-imaged^22^ mean distance matrices, as well as the model and Hi-C^53^ bulk contact matrices, have a high correlation (respectively, *r*_s_ = 0.96 and *r*_s_ = 0.94) (Fig. 2d and Supplementary Fig. 4a,b,d). Importantly, as in the other loci considered (see below), the in silico SPRITE and GAM average matrices also faithfully represent the mean distance data (correlation, *r*_s_ = −0.98 and *r*_s_ = −0.99, respectively; Supplementary Fig. 4c). As an additional check, to compare the distance matrices of the imaged data, of the SBS and of the SAW models, we computed their genomic-distance-corrected Pearson correlation coefficient, *r*′ (Methods). We found (Supplementary Fig. 5a–c and Methods) that the mean distance matrix of the SAW model is featureless, with no TADs or patterns, and it has a much lower correlation with the experimental one (*r*′ = 0.32) than the SBS model mean distance matrix (*r*′ = 0.84). Finally, we extended the comparison to single-molecule distance data. We computed the distribution of *r*′ values between the pairs experiment–experiment, experiment–SBS and experiment–SAW single-molecule distance matrices, and found that while the first and second distributions are not statistically distinguishable (two-sided Mann–Whitney *U*-test *P* = 0.19), the experiment–SAW distribution is clearly different (*P* = 0; Supplementary Fig. 5d).

Taken together, the agreement between model and experiments provides a validation of our polymer model because its 3D structures inferred from Hi-C data accurately recapitulate independent SPRITE, GAM and microscopy data, even at the single-molecule level, consistently across different experiments, loci and cell types. The consistent agreement also shows that our in silico approach has no particular biases favoring Hi-C, SPRITE or GAM.

### In silico bulk Hi-C, SPRITE and GAM data describe the benchmark average distance matrix

**Fig. 3:**
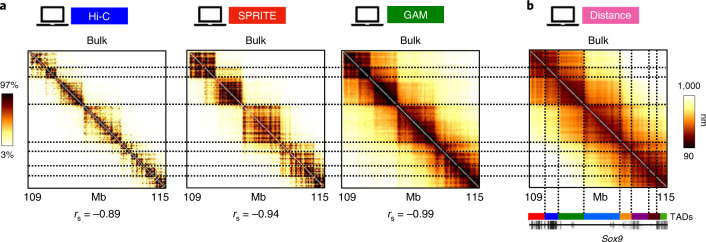
Bulk Hi-C, SPRITE and GAM data are faithful overall to average 3D distances. **a**, In silico bulk Hi-C, SPRITE and GAM maps of the *Sox9* locus are shown and compared to the average 3D distance matrix in **b** of the known single-molecule 3D conformations of the locus model. The color scale represents the percentiles in each dataset. Spearman correlation coefficients are reported, on the bottom, between each contact map and the average 3D distance matrix (Pearson and HiCRep are reported in Supplementary Table 1b). **b**, Average 3D distance matrix derived from the ensemble of in silico model single-molecule 3D conformations.

Next, we investigated how well in silico Hi-C, SPRITE and GAM data reflect the spatial architecture of the underlying ensemble of model conformations. In the case study of the *Sox9* locus, we computed the average distance matrix of the known 3D structures and compared it with in silico Hi-C, SPRITE and GAM bulk data—that is, averages over in silico cells (Fig. 3). The absolute values of Spearman correlation coefficients (as well as of Pearson and HiCRep correlations; Supplementary Table 1b) of the three methods with the average distance matrix are all high (*r*_s_ < −0.89; values are negative because large physical distances correspond to small contact frequencies).

Interestingly, the patterns visible in the in silico Hi-C, SPRITE and GAM bulk data are similar to each other, albeit GAM better highlights longer-range contacts between TADs (Fig. 3a). In particular, all three in silico methods identify the known TADs of the locus^9,10,19^ (Fig. 3b, different colors in the bottom bar). Additionally, Hi-C, SPRITE and GAM data match the domain-like patterns of the average 3D distance matrix, which represent the typical folding of the reference ensemble of model conformations (Fig. 3b). Similar results are found for the murine *HoxD* and *Epha4* loci and, as already stated, the human locus (Supplementary Figs. 1c,d, 2b,c and 4b,c).

Taken together, our results support the view that bulk data from Hi-C, SPRITE and GAM are faithful to the overall spatial structure of the underlying 3D conformations in murine and human loci and, albeit that differences exist, they provide comparable information on average distances.

### Stochasticity of single-cell data reflects the intrinsic variability of single-molecule 3D conformations

Whereas bulk Hi-C data are comparatively similar across replicates, single-cell data exhibit strong variability^54–58^. Here we explore two sources of such variability: limited detection efficiency and, importantly, inherent differences across single-molecule conformations of chromatin.

**Fig. 4:**
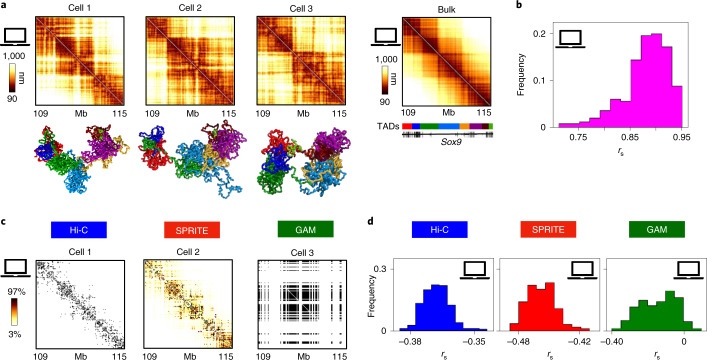
Stochasticity of single-cell contact maps reflects the intrinsic variability of single-molecule 3D conformations. **a**, In silico single-cell distance maps (top) and average distance matrix (top right) of the *Sox9* locus are shown. The variability of the corresponding single-molecule conformations of the model is represented in the bottom of the panel while the color scheme reflects the colors of the TADs of the locus^19^, shown in the color bar. **b**, The distribution of Spearman correlations between in silico single-cell distance maps and the average distance map is shown (mean value *r*_s_ = 0.88). Mean Pearson and HiCRep correlations are reported in Supplementary Table 1c. **c**, In silico single-cell Hi-C, SPRITE and GAM contact maps corresponding to the first of the three in silico cells in **a** are shown (color scale indicates the percentiles of each map). Here, because the in silico efficiency is set to 1, all contacts are captured in Hi-C or SPRITE and all segregated windows are detected in GAM. **d**, The distribution is shown of Spearman correlation coefficients between in silico single-cell contact matrices at efficiency = 1 and their corresponding in silico single-cell distance matrices.

To investigate the impact on contact maps of the structural variability of single molecules, we discuss first the ideal case of in silico experiments where the efficiency is set to 100%. Consistent with single-cell imaging data^21–24^, single-molecule conformations vary widely in the ensemble of model 3D structures^28^ (Fig. 4a, bottom) and their single-cell distance matrices (Fig. 4a, top) have broadly varying Spearman correlations with the average distance matrix (Fig. 4b; mean *r*_s_ = 0.88; Supplementary Table 1c). Additionally, the correlation of an in silico single-cell Hi-C, SPRITE or GAM contact map (Fig. 4c) with its corresponding single-cell distance matrix is much lower than in the case of the bulk data previously discussed, with, on average, *r*_s_ = −0.37 and *r*_s_ = −0.46 for, respectively, in silico Hi-C and SPRITE (Fig. 4d and Supplementary Table 1d). As expected, for GAM the correlation between single-cell maps is even lower (average *r*_s_ = −0.15) and its distribution much broader, in the range −0.4 < *r*_s_ < 0. That is also a consequence of the different experimental procedures: while a single-cell in silico Hi-C or SPRITE experiment returns the contacts measured over an entire in silico nucleus—that is, two independent polymer structures representing the alleles—a single-cell in silico GAM experiment probes the polymer content of only a single slice of an in silico nucleus—that is, a tiny fraction of the two polymers.

Contact data from single-cell experiments become further deteriorated at lower values of detection efficiency and have worse correlations with the corresponding single-cell distance maps (Supplementary Table 1e). Consequently, the variability of replicates from in silico single-cell experiments increases and the correlation between their contact maps correspondingly decreases. For example, for an efficiency of 0.5, we found that the average correlation between in silico single-cell replicates is around *r*_s_ = 0.2, 0.4 and 0.1 for Hi-C, SPRITE and GAM maps, respectively. While the impact of limited efficiency on contact maps is systematically investigated in the following section, here we stress that the values of correlation measured between single-cell replicates are consistent with those reported in real experimental studies. For example, in real single-cell experiments in CD4 T_H_1 cells with an efficiency of approximately 0.025 (ref. ^55^), the average Spearman correlation between different Hi-C maps of the *Sox9* locus was found to be *r*_s_ = 0.01, which is numerically equal to the value found for the same efficiency in our model of mESC (Methods and Supplementary Fig. 6a).

To summarize, the variability of in silico single-cell Hi-C, SPRITE and GAM data reflects the intrinsic structural differences across chromatin single molecules, and they are less faithful than bulk data to the corresponding single-cell distances even in the ideal case of 100% detection efficiency. Lower efficiencies further increase fluctuations, to the point where single-cell replicates can have correlations well below 0.1 at realistic efficiencies, hence explaining the variability of single-cell experiments^9,54–57^.

### The number of cells required for replicate reproducibility differs among Hi-C, SPRITE and GAM

**Fig. 5:**
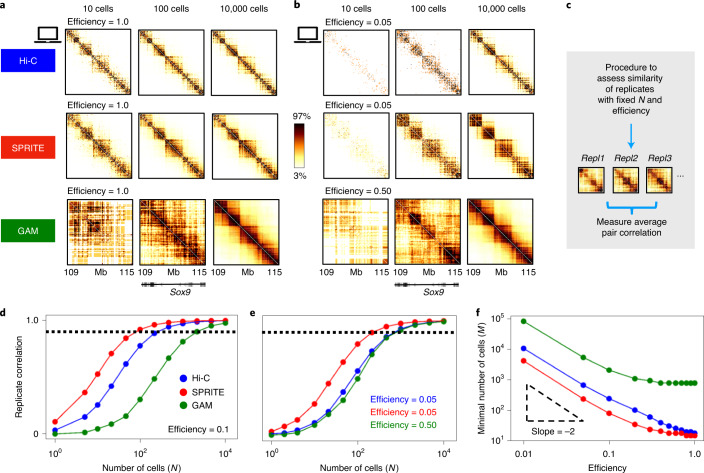
The number of cells required for replicate reproducibility differs among Hi-C, SPRITE and GAM. **a**, In silico Hi-C, SPRITE and GAM contact maps of the *Sox9* locus under different numbers of in silico cells (*N*) (at efficiency = 1) are shown. Color scale indicates the percentiles of each map. **b**, In silico efficiencies similar to those of real experiments are set—that is, 0.05 for Hi-C and SPRITE and 0.5 for GAM. **c**, The minimal number of cells (*M*) having a reproducible output map is defined as the value of *N* where the average Pearson correlation between replicates crosses the threshold *r*_t_ = 0.9. **d**,**e**, Pearson correlation between replicate experiments as a function of *N* for Hi-C, SPRITE and GAM at a given efficiency (0.1) (**d**) and for efficiencies corresponding to typical experimental values (**e**). Here, the Hi-C and SPRITE curves are at 0.05 efficiency, the GAM curve at 0.5. The dashed line denotes the threshold correlation value *r*_t_ = 0.9. **f**, The value of *M* is shown for Hi-C, SPRITE and GAM as a function of efficiency.

The quality of in silico Hi-C, SPRITE and GAM contact maps improves when the number of in silico cells (*N*) in the experiment increases (Fig. 5a). Figure 5b shows, for example, the effect of *N* in the case of efficiencies comparable to typical experimental values: we set the Hi-C efficiency to 0.05, taken as an upper limit of values reported in recent studies^54–56,59^; the same value is used as an estimate of the efficiency for SPRITE. Because the experimental efficiency in GAM^9^ is roughly one order of magnitude larger than in Hi-C and SPRITE, in the example shown we used an in silico GAM efficiency of 0.5 (Methods).

Importantly we checked that, in the large *N* limit, (1) the contact matrices overall are not dependent on the efficiency value considered and (2) the average over a large number of cells compensates for reduced efficiencies (see below and Methods). Indeed, as shown in Fig. 5a,b, the patterns of contact matrices become sharper and stabilize when *N* becomes sufficiently large, as also observed in experimental investigations^55,60^. However, our data show that the threshold value of *N* to reach saturation is strongly dependent on the efficiency level and varies with different technologies.

We aimed to identify the minimal number of cells that, at a given efficiency, is required for replicate in silico experiments to return similar outputs—that is, to approach the bulk limit. To measure the similarity of pairs of identical experiments in the *Sox9* locus (all with the same *N* and efficiency; Fig. 5c), we computed the average Pearson correlation between their contact maps (Fig. 5d,e; Spearman and HiCRep correlations returned analogous results; Supplementary Fig. 7). The correlation grows when *N* is increased and plateaus to 1 at the large *N* limit (Fig. 5d,e), independently of the efficiency of the in silico experiment. For each given efficiency, we heuristically defined the minimal number of cells, *M*, required for statistically reproducible results across replicates as the value of *N* where the correlation grows larger than a given threshold, *r*_t_ = 0.9 (Methods). Interestingly, we found that *M* is different in the different technologies: for example, if the efficiency is 0.1 we found that *M* is 200, 100 and 2,000 for Hi-C, SPRITE and GAM, respectively (Fig. 5d). Figure 5e shows the correlation between replicates at varying *N* obtained for efficiencies close to those reported in real Hi-C, SPRITE and GAM experiments—that is, as specified above, 0.05 for Hi-C and SPRITE and 0.5 for GAM: in those cases, *M* is approximately 650, 250 and 800, respectively. Additionally, we checked that our estimates of *M* compare well against available experimental estimations (Methods and Supplementary Fig. 6b).

Next, we systematically investigated how the quality of in silico data is affected by the efficiency of the experiment (Supplementary Fig. 11). We found that the number of cells required for replicate similarity (*M*) is strongly dependent on efficiency (Fig. 5f): *M* diverges approximately as an inverse squared power law as the efficiency becomes small. In other words, halving the efficiency requires the quadrupling of cell number to achieve the same quality levels. In general, we find that *M* for SPRITE is two times smaller than the corresponding value for Hi-C and one order of magnitude smaller than for GAM. Additionally our investigation shows that, even in the ideal case of efficiency = 1, single-cell replicates have below-threshold correlations because *M* > 10 even for SPRITE, due to the intrinsic variability of single-molecule 3D structures, as reported above.

Similar results were found for the murine *HoxD*, *Epha4* and human HCT116 loci (Supplementary Figs. 8a–d, 9a–d and 10a–d). The sets of in silico single-molecule 3D structures employed in all our analyses were produced using polymer models inferred from Hi-C data^25–28^. However, for the *Sox9* locus we tested that our results remained unchanged overall also when the polymer model of the locus is inferred from GAM contact maps rather than from Hi-C^48^ (Supplementary Figs. 13 and 14a–d and Methods). Additionally, to assess the general validity of our analyses, we applied the in silico approach to 3D conformations of a toy block-copolymer, unrelated to real chromatin loci, finding similar results (Supplementary Fig. 15 and Methods). Finally, we checked that the above definition and features of *M* can be fully grounded on the central limit theorem (CLT; Methods and Supplementary Fig. 12).

GAM cosegregation data, as mentioned before, include both random and nonrandom cosegregation events (that is, specific interactions) that can be dissected by the use of statistical methods such as SLICE^9^ (Methods). Hence, we investigated the performance of SLICE on in silico GAM data when both *N* and efficiency varied (Supplementary Fig. 16). SLICE returns, in particular, the single-cell interaction probability of pairs, and multiplets, of DNA sites^9^. We found that SLICE bulk interaction probabilities are faithful to the known average distance matrix (*r* = −0.95, *r*_s_ = −1.00, scc = −0.99; Supplementary Fig. 16a,b) and the SLICE matrices behave with both *N* and efficiency as found for GAM contact maps. Because by definition SLICE specifically detects significant interactions, however, we found that the average number of in silico cells (*M*) needed to return statistically reproducible results across replicates is approximately half that required for GAM alone under the same conditions (Supplementary Fig. 16c–e). For a realistic efficiency of 0.5, for example, we found that *M* = 400 for SLICE whereas *M* = 800 for GAM. In that respect, SLICE can be employed as a useful tool to enhance the performance of GAM, especially in applications where the number of available cells is small, such as in the analysis of sample tissues or biopsies.

Our findings illustrate how the level of variability of in silico contact matrices is affected by both the *N* value and experimental efficiency, and how different technologies perform under different situations. Consistent with the CLT, the number of cells required for replicate similarity (*M*) grows as an inverse squared power law as efficiency decreases.

### Noise-to-signal levels vary differently with genomic distance in Hi-C, SPRITE and GAM

**Fig. 6:**
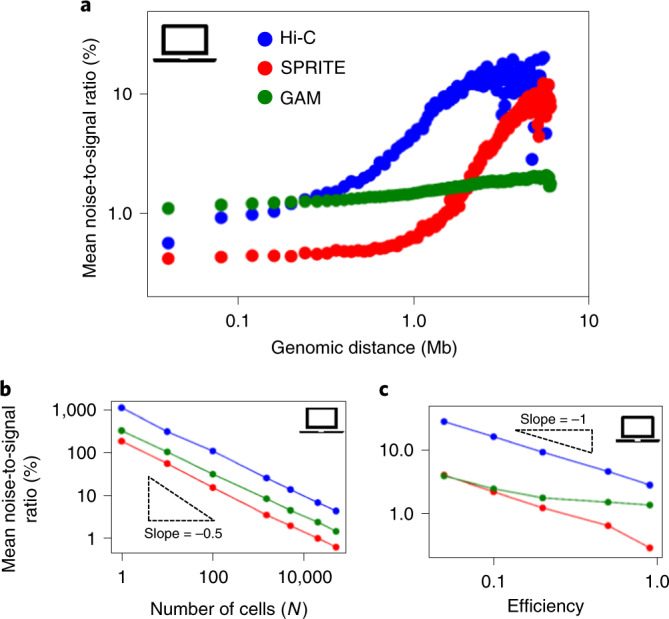
Noise-to-signal levels vary differently with genomic distance in Hi-C, SPRITE and GAM. **a**, The mean noise-to-signal ratio (*σ*/*μ)* of a contact map, for *N* = 50,000 and efficiency = 0.5, is dependent on the genomic separation considered. **b**, For a genomic distance of 1 Mb and efficiency of 0.5, *σ*/*μ* decreases with *N* as an inverse square root, as expected from the CLT. **c**, For a genomic distance of 1 Mb and *N* = 50,000, *σ*/*μ* increases approximately as an inverse power law when efficiency is reduced.

Finally, we investigated the noise-to-signal level of the entries of contact matrices and how it varies with genomic separation, with *N* and with the efficiency of in silico experiments. For each entry of a contact map, the noise-to-signal ratio is defined as the ratio of the standard deviation, *σ*, to the mean value, *μ*, across replicate experiments under the same conditions. For a given *N* and efficiency, we observed that the average noise-to-signal ratio, *σ*/*μ*, is strongly dependent on genomic distance (Fig. 6a and Methods). In the *Sox9* locus, we found for both Hi-C and SPRITE that *σ*/*μ* increases by more than one order of magnitude as genomic separation increases from 50 kb to 5 Mb. In particular, there is a steep increase above the 1-Mb level. SPRITE has the lowest *σ*/*μ* ratio at genomic scales below 1 Mb but, interestingly, GAM has an overall lower varying noise-to-signal level, especially at large genomic separations (>1 Mb) where it is almost one order of magnitude lower than Hi-C and SPRITE.

At a given genomic distance and efficiency, as expected, the noise-to-signal ratio decreases as *N* increases (Fig. 6b). Consistent with the CLT, it follows an inverse squared power law in *N* (that is, *N*^−1/2^). One consequence of such a scaling behavior is that single-cell (*N* = 1) contact maps become highly noisy at large genomic separations. For example, at the 1-Mb scale and for a detection efficiency of 0.5, the noise-to-signal ratio for *N* = 1 is >100% for all three methods, with Hi-C having the largest fluctuations (*σ*/*μ* > 1,000%). As expected, the noise-to-signal ratio is also strongly affected by experimental efficiency (Fig. 6c): in brief we find that, in our in silico study, for a given genomic distance and a given *N*, *σ*/*μ* decreases roughly as an inverse power law of efficiency.

Similar results were obtained for the murine *HoxD*, *Epha4* and human HCT116 loci (Supplementary Figs. 8e–g, 9e–g and 10e–g). Finally, we extended all the analyses done on the model of the *Sox9* locus derived from Hi-C data to the polymer model derived from GAM data^48^ and acquired results fully consistent with our previous findings, supporting the broader validity of our approach (Supplementary Fig. 14e–g).

## Discussion

Hi-C, SPRITE and GAM are powerful technologies utilized to probe DNA contacts genome wide. We discuss a quantitative benchmark to assess how well these different methods represent the 3D structure of the genome and how they perform relative to each other under different experimental conditions. Our approach is based on computer simulations of their performance in capturing the architecture of a known set of polymer 3D structures. We focused on the models of regions around genes *Sox9* and *HoxD* in murine ES cells, around the *Epha4* gene in CHLX-12 cells and in human HCT116 cells, as well as on a toy block-copolymer model. We analyzed in silico Hi-C, SPRITE and GAM with different experimental parameters including the cell numbers considered, detection efficiency and genomic separation scales (Table 1). There is a consistent agreement between independent Hi-C, SPRITE and GAM data and our in silico models across the studied loci and cell types (Fig. 1). Additionally, in the human HCT116 locus for which single-cell microscopy data are available^22^, we verified that the model polymer conformations provide a bona fide representation of the experimental single-molecule 3D structures (Fig. 2).

**Table 1:** Summary of the performance of in silico Hi-C, SPRITE and GAM under identical conditions

Method	Faithfulness to 3D structure		Replicate similarity versus cell number	Noise versus detection efficiency	
	Bulk data	Single-cell		<1 Mb	>1 Mb
Hi-C	+++	++	++	++	+
SPRITE	+++	+++	+++	++	++
GAM	+++	+	+	+	+++

We find that in silico Hi-C, SPRITE and GAM bulk contact data, as well as SLICE interaction probabilities, faithfully represent the known spatial conformations of model polymers (Fig. 3). Single-cell contact data reflect the intrinsic broad structural variability of chromatin single molecules and are much less faithful to their corresponding single-cell distance matrices (Fig. 4). Because single-cell GAM captures a single slice of a nucleus rather than the entire nucleus, its fluctuations are even stronger than in single-cell Hi-C and SPRITE.

The minimal number of in silico cells (*M*) to be considered in an experiment for replicates to return sufficiently similar contact patterns (Fig. 5) increases approximately as an inverse squared power as the efficiency of the experiment decreases. For equal conditions, *M* varies in different technologies (Fig. 5): SPRITE has the lowest *M* and so performs better on samples with a small number of cells; GAM has the highest value, but its *M* is reduced if GAM is employed in combination with SLICE, a statistical tool utilized to extract nonrandom contacts. In real applications the efficiency varies across the three methods and the corresponding values of *M* can become similar. For example, experimental estimates of the efficiency of Hi-C are around^59^ 0.05 and are similar for SPRITE, while for GAM it is about^9^ 0.5. Under those conditions we find that *M* is around 650, 250 and 800 for Hi-C, SPRITE and GAM, respectively; additionally, when GAM is combined with SLICE, *M* becomes approximately 400.

The noise-to-signal ratio in contact maps is affected by the cell number employed in in silico experiments, and grows approximately as a power law by decreasing efficiency (Fig. 6). Genomic distance also impacts the noise-to-signal ratio (Fig. 6): for identical conditions, GAM is less noise sensitive at large genomic separation (say, >1 Mb).

Overall, our computational analyses are consistent across the investigated cases (models of both real loci and toy models), supporting the view that quantitative comparison of the performance of in silico Hi-C, SPRITE and GAM has a more general validity. Albeit simplified, the resulting picture can thus help in the rational design of real-world applications of those technologies for specific purposes and in different contexts.

## Methods

### The SBS model

The SBS model^38,47^ describes a chromatin segment as a self-avoiding polymer chain comprising beads interacting with diffusing molecules, called binders. Along the polymer chain, binders can attractively interact with beads that act as binding sites. The interaction is specific, such that different types of binding site interact only with their associated binders. The model can be visually represented as a chain with beads of different colors, where only same-color bead–binder pairs interact. Because the interaction is multivalent, a binder can attach to multiple beads. The number of colors (that is, types of binding site), their location along the polymer chain and the concentration of binders regulate the folding dynamics and equilibrium configurations of an SBS polymer^25,38^.

### Inference of the SBS model of a DNA locus from experimental data

The SBS model of a given genomic locus is a polymer with a convenient set of binding sites (colors) suitably arranged along the chain. To infer the genomic positions of different colors, a machine learning method, named PRISMR^25,27^ is employed. Briefly, PRISMR finds the SBS polymer that best reproduces a given input contact matrix, without any previous assumptions, based only on the principles of polymer physics^27^. Input contact matrices—as Hi-C^27^ or GAM^48^—describing a genomic region of length *L* base pairs (bp) at resolution res are *N*_bin_ × *N*_bin_ sized, where *N*_bin_ = *L*/res is the number of DNA windows (bins) in the region considered. In general, the best SBS polymer describing such a DNA region will be composed by *N*_bead_ *=* *N*_bin_ *×* *n* beads, where *n* accounts for the presence of different binding sites in a bin and is given by PRISMR.

Once the polymer model of the locus of interest is found, its 3D conformation is derived by a massive parallel MD computer simulation at thermodynamic equilibrium. This is repeated for several independent runs to yield an entire ensemble of single-molecule 3D equilibrium structures. All MD simulations are carried out using the free software LAMMPS^61^, with interaction potentials previously established in the literature^62^. Full details of the models employed and simulation parameters can be found in the referenced papers. This entire approach has been successfully used to reproduce experimental data (FISH, Hi-C or GAM)^25,28,38,48^, and provided correct predictions on the impact of mutations over 3D DNA organization^27^.

### Model 3D structures of murine *Sox9*, *HoxD* and *Epha4* and human HCT116 cell loci

The SBS polymer model of the murine *Sox9* locus (chr11:109–115 Mb, mm9) was inferred^25^ by PRISMR from mESC Hi-C data at 40-kb resolution^19^, and consists of 2,250 beads and 15 types of binding site (colors). The ensemble of its 3D structures derived by MD is composed of 500 configurations. Full details on MD simulation parameters are previously described^25^.

To check the robustness of the approach, we also employed a second ensemble of SBS 3D structures describing the same genomic region but derived^48^ from GAM mESC 40-kb cosegregation data^9^. The polymer consists of *N*_bead_ = 2,250 and 15 colors and the ensemble comprises 450 equilibrium structures. Details of the MD simulation are previously published^48^ and the parameters used are the same as previously described^25^.

The SBS polymer model for the murine *HoxD* locus (chr2:71–78 Mb, mm9) was derived^26^ by PRISMR from mESC Hi-C data at 40-kb resolution^19^. The polymer comprises *N*_bead_ = 2,100, with 12 colors; the ensemble comprises 500 equilibrium configurations. Full details of the MD simulation are previously reported^26^.

In regard to the murine *Epha4* locus (chr1:73–79 Mb, mm9), the polymer model was obtained^27^ by PRISMR applied to CHLX-12-cell in situ Hi-C data at 10-kb resolution^4^. Here, the polymer is *N*_bead_ = 12,600 beads long, with 21 colors. The ensemble comprises 500 equilibrium configurations. Full details of MD simulation parameters are previously reported^27^.

The SBS model of the human HCT116 locus (chr21:34.6–37.1 Mb, hg38) was inferred^28^ from Hi-C data at 30-kb resolution^53^. The polymer consists of 830 beads and four types of binding site, and MD simulations provided 1,000 3D equilibrium conformations. All further details of MD simulations can be found in previous work^28^.

### 3D structures of the block-copolymer toy model

To test our algorithms and results, we also employed a toy SBS block-copolymer model unrelated to real DNA loci. We considered a polymer chain of 210 beads with only three different colors (Supplementary Fig. 15a); two of those colors (green and red) were arranged in separate blocks. Visually, we colored in green the first block and in red the second. In addition, to permit specific contacts across both blocks, we introduced a single bead of a third color, represented in blue, at approximately the middle position of each color block. Potentials and parameters used for MD simulations are the same as previously described^25^ and, in particular, the interaction energy scale was set at 9*k*_B_*T* for the red and green beads and at 12*k*_B_*T* for the blue beads, with *k*_B_ being the Boltzmann constant and temperature *T* = 300 K. The interaction range was set at 1.3*σ* for the red and green beads and 1.5*σ* for the blue, where *σ* is the bead diameter. Four binders of the blue type and 88 each of the red and green were employed to make the polymer fold. At equilibrium, the green and red blocks segregate separately forming two globular domains while the isolated blue beads produce a long-range contact stretching out from their respective blocks (Supplementary Fig. 15a,b). The ensemble is composed of 150 structures. Here, a bin is assumed to be formed by a single bead, such that *N*_bin_ *=* *N*_bead_.

### GAM data

GAM data were obtained from mouse ESCs (clone F123), a male cell line derived from an F1 *Mus musculus castaneus* *×* *S129/SvJae*^63^, kindly provided by B. Ren (University of California San Diego, San Diego, CA, USA). Normalized contact matrices from individual nuclear profiles (NPs) of the F123 cell line at 40 kb were obtained from the 4D Nucleome Consortium^52^ and are available on the 4D Nucleome data portal under accession no. 4DNFIFBSQ1EO. Normalization of cosegregation frequencies was performed using pointwise mutual information (PMI). The PMI of genomic windows *i* and *j* describes the difference between the probability of both windows being found in the same NP (that is, their joint distribution) and their individual distributions across all NPs. PMI assumes that finding one window is independent of finding the second. Specifically, for windows *i* and *j*, the value of PMI is

$${\mathrm{PMI}} = \log \left( {\frac{{p({{i,j}})}}{{p\left({{ i}} \right)p({{j}})}}} \right),$$

where *p*(*i*) is the frequency whereby window *i* was found across the NPs (*p*(*j*) is analogously defined) and *p*(*i*,*j*) is the frequency that the two windows are found together across the NPs. PMI is then normalized (NPMI) by bounding between −1 and 1 as follows:

$${\mathrm{NPMI}} = - \frac{{{\mathrm{PMI}}}}{{\log \;p\left( {{{i,j}}} \right)}}.$$

### Comparison between experimental and in silico contact matrices

Quantitative comparisons between in silico and experimental matrices were performed computing the Spearman (*r*_S_) and Pearson (*r*) correlation coefficients, and the stratum adjusted correlation coefficient (scc) from the HiCRep method, developed specifically for Hi-C data^51^. The experimental data used for comparisons (Fig. 1b, Supplementary Table 1a and Supplementary Fig. 13a) with the in silico data of the *Sox9* region are Hi-C^19^ and SPRITE^10^ in mESC and GAM from the F123-cell 1,122 × 1NP dataset. All datasets are at 40-kb resolution. For the *HoxD* locus, the experimental data used (Supplementary Fig. 1a) are the same as for the *Sox9* locus. For the *Epha4* locus, the Hi-C data^4^ in mouse CHLX-12 cells at 10 kb were employed for comparison with our in silico Hi-C map (Supplementary Fig. 2a). All datasets used are aligned to the mm9 assembly. Finally, for the locus in the human HCT116 cell line, we considered the Hi-C data^53^ and the distance matrices from imaging data^22^, at 30 kb and aligned to the assembly hg38 (Fig. 2d and Supplementary Fig. 4).

### In silico distance matrices

To compute the distance matrix of a given polymer model, we calculate the Euclidean spatial distance between all pairs of beads, obtaining a two-dimensional (2D) matrix *N*_bead_ × *N*_bead_. Then, we coarse-grain it by averaging over all entries belonging to the same pair of bins, to acquire a distance map *N*_bin_ × *N*_bin_. Bulk distance matrices (shown in Figs. 2d and 3b and Supplementary Figs. 1c, 2b, 4b,13b and 15b) are obtained by averaging over all the ensemble of polymer structures. Conversely, single-cell distance matrices (Fig. 4a) are an average over the single pair of polymers comprising a simulated cell.

The unit length of distances in the polymer models is *σ*—that is, the diameter of a bead in our MD simulations. We set the value of *σ* based on estimates of the chromatin compaction factor, as in previously published works^27,39,64^. For example, in the case of *Sox9*, the genomic content per bead is *g* = *L*/*N*_bead_ = ∼2,667 bp, with *L* = 6 Mb (locus genomic length) and *N*_bead_ = 2,250 (number of polymer beads). We used a chromatin compaction factor of 70 bp nm^–1^—that is, an average value between the 30-nm fiber and the naked DNA^39,65^, which returned *σ* = 38 nm. Analogously for the *Epha4* polymer model, that is also 6 Mb in length. For the *HoxD* and HCT116 locus polymer models (7.0 and 2.5 Mb in length, respectively), the same calculations yielded *σ* = 48 nm and *σ* = 43 nm, respectively. In the toy block-copolymer model we express distances simply in units of *σ* (Supplementary Fig. 15).

### In silico simulations of Hi-C, SPRITE and GAM

The codes used to implement in silico Hi-C, SPRITE and GAM take as input the spatial coordinates of the beads of our polymer 3D structures. To take into account the two alleles present in each single cell, we explicitly consider pairs of independent structures in our simulations. By repeating the algorithms over different, randomly selected polymer pairs, we simulate experiments carried over a population of cells. All codes are written in the C programming language.

### In silico Hi-C

For in silico Hi-C experiments we implemented a proxy of the key steps of a Hi-C protocol^2,4,54,55,57^—that is, crosslinking, digestion, biotinylation, ligation and contact matrix generation, as described in detail in the following sections. In particular we applied those steps separately to each in silico cell, as in a real single-cell Hi-C experiment.

#### Crosslinking

During real Hi-C crosslinking, DNA contacting sites are bound together with formaldehyde to fix the overall 3D structure. Formaldehyde binds to DNA–protein complexes and consequently fixes DNA sites that are linked by protein bridges^66^. In our SBS polymers only same-colored beads interact with each other through a binder and only if they are closer than a threshold distance *d*, fixed by the interaction energy cutoff^25^. Thus, for in silico Hi-C we crosslink beads of the same color and that are closer than *d*. This is done with efficiency *p*_c_, simulating the experimental one. To identify the sets of crosslinked beads, a customized version of the DBSCAN clustering algorithm^67^ is employed.

#### Digestion

After crosslinking, DNA is digested—that is, cut into fragments. In standard Hi-C experiments, digestion fragments have a median length in the range from few hundreds of base pairs to several kilobases, depending on the restriction enzyme used^55^. Importantly, in the SBS models of the *Sox9*, *HoxD*, *Epha4* and human loci, because the genomic content of each bead falls within that range a single polymer bead is a good representation of the average digestion fragment. Thus, we implement digestion by splitting the polymer chain into its own individual beads. As result, we acquire a set of independent clusters consisting of crosslinked beads.

#### Biotinylation

The next step is biotinylation, where DNA fragments in each crosslinked cluster are marked with biotin. Unmarked fragments cannot be detected in Hi-C. In our algorithm, biotinylation is implemented by removing beads from their clusters with probability 1 – *p*_b_, with *p*_b_ modeling biotinylation efficiency.

#### Ligation

In Hi-C, crosslinked and biotinylated pairs of fragments are randomly linked, forming contacting pairs. This is implemented by random selection of pairs of beads from the same crosslinked cluster within threshold distance, *d*. To account for experimental ligation efficiency, each selected bead is ligated with a probability of only *p*_l_, and is otherwise discarded.

#### Contact matrix generation

Next in Hi-C, ligated fragments are sequenced and a contact is counted between their corresponding bins, eventually producing a contact matrix *N*_bin_ × *N*_bin_. Similarly, in our algorithm we produce a 2D *N*_bin_ × *N*_bin_ matrix. For each polymer structure in input, ligated beads are counted as a contact with given detection probability *p*_d_—modeling the sequencing efficiency of real experiments—and their corresponding matrix entry is incremented by 1. The procedure is iterated over the *N* simulated cells, and the final in silico matrix yields the total count of contacts between each possible pair of bins.

### In silico SPRITE

For SPRITE, we implemented the main steps of its protocol^10^—crosslinking, digestion, split-pool tagging and contact matrix generation.

#### Crosslinking

In SPRITE experiments, crosslinking is carried out as in Hi-C^10^, so the same procedure described above for our in silico Hi-C is employed.

#### Digestion

After crosslinking, in SPRITE experiments DNA is fragmented first by sonication and then by DNAse digestion, resulting in a collection of crosslinked fragments of approximately 150–1,000 bp (ref. ^10^), similar to restriction fragments produced by digestion in Hi-C. Hence, we implement SPRITE chromatin digestion with an algorithm analogous to that used for in silico Hi-C.

#### Split-pool tagging

The split-pool tagging procedure allows identification of DNA fragments belonging to the same crosslinked cluster, because they are uniquely barcoded and all DNA fragments belonging to the same cluster are associated with a specific tag sequence^10^. In our in silico procedure, because the beads composing a given cluster are known, an explicit split-pool tagging implementation is not required. However, to take into account the fact that in real experiments some fragments may not be tagged successfully, we remove beads from their clusters with probability 1 – *p*_s_.

#### Contact matrix production

Experimentally, fragments with the same barcode are sequenced and assigned to their corresponding genomic windows—that is, the bins comprising the contact map. In this way it is possible to define those bins associated with a fixed cluster. A contact is then counted for every possible pair of bins associated with a cluster. The count is weighted by a corrective factor 2/*n*, taking into account the size of the cluster, with *n* the number of fragments in the cluster^10^. In our in silico procedure, because each fragment is represented by a polymer bead, we assign a count to a bin pair if at least one bead from each bin is found in the same cluster. As with in silico Hi-C, each bead is detected with only given probability *p*_d_, modeling sequencing efficiency. Each contact count over the population of simulated cells is then multiplied by its weight, 2/*n*, and finally collected in a *N*_bin_ × *N*_bin_ matrix.

### In silico GAM

In GAM experiments, a nuclear slice is cut in random orientation from a cell nucleus, the DNA sites in the slice are sequenced and their co-occurrence in a collection of slices measured to construct a GAM cosegregation matrix^9^. In our algorithm, nuclear slicing and cosegregation matrix generation were implemented as follows.

#### Slice cutting

We model a cell nucleus as a sphere containing two different, randomly placed, polymer structures of the locus of interest, to take into account both alleles present in the considered cell type. For each in silico cell we generate a randomly oriented slice within the sphere, and all polymer beads inside are counted as cosegregating. The in silico nuclear radius and slice thickness are set, in units of *σ* (polymer chain bead size), to match the scales of the experimental values in the considered cell types^9,25,68,69^. The simulated slices may not contain the specific locus of interest, as in a real GAM experiment, where cellular slices contain only a fraction of the nucleus. To account for experimental detection and sequencing efficiencies, beads inside a simulated slice are counted only with certain probability, *p*_d_.

#### Cosegregation matrix production

In GAM, bins found in the same slice are detected with a given efficiency and counted as cosegregating. Cosegregation frequencies are then arranged in a 2D *N*_bin_ × *N*_bin_ matrix. Similarly, in our algorithm, we build a 2D *N*_bin_ × *N*_bin_-sized matrix. Reflecting the process of calling positive windows in experimental GAM data, if at least one single bead belonging to a bin is present in a simulated slice we consider the whole bin to be inside it. We therefore count all possible pairs of bins found in a slice and add that to the corresponding entry in the cosegregation matrix. We finally normalize the matrix by the number of slices employed, to generate cosegregation frequencies.

#### SLICE

We applied the statistical tool SLICE over in silico cosegregation data to determine its performance (Main Text and Supplementary Fig. 16). To do so, we considered the SLICE model statistics previously developed^9^ and implemented a version of the SLICE algorithm customized for the application on the in silico data of genomic loci.

### The efficiency of in silico experiments

Each step of the in silico Hi-C algorithm has a specific efficiency: the probability *p*_c_ for the inclusion of a bead in a crosslinked cluster, the probability *p*_b_ for the survival of a bead in a cluster, the probability *p*_l_ for ligation of a bead and the probability *p*_d_ for the detection of a ligated bead. Similarly, each step of in silico SPRITE and GAM has limited efficiency. By construction, in our algorithms the different steps are all independent and hence the overall in silico efficiency, *ε*, that we discuss in Main Text is simply the product of single-step efficiencies. For example, for Hi-C, different values of *p*_c_, *p*_b_, *p*_l_ and *p*_d_ corresponding to a given *ε* yield the same average output. The same holds for SPRITE and GAM.

The in silico overall efficiency refers to the probability of the detection of a polymer bead in our Hi-C, SPRITE and GAM algorithms, and can be mapped onto the corresponding experimental overall efficiency. For Hi-C, since the average length of a digestion fragment equals the size of a polymer bead in our models, in silico overall efficiency can be assumed to be a good proxy for single-cell efficiency in a real Hi-C experiment, and analogously for SPRITE. In the case of GAM, experimental efficiency is computed via the SLICE algorithm^9^ and provides the probability for detection of a DNA window (that is, a bin) present in a physical slice. The link between in silico and experimental bead efficiency can be derived as follows. In a simulated GAM experiment, if *k* beads of a bin fall within a slice, the probability of detection of such a bin is 1 − (1 − *ε*)^*k*^. Averaging over all the permitted values of *k*, the following approximate relation links bead and GAM efficiency:

$$\varepsilon _{{\mathrm{{\scriptsize{{\scriptsize{GAM}}}}}}} = 1 - \frac{{1 - \varepsilon }}{\varepsilon }\frac{{1 - \left( {1 - \varepsilon } \right)^{2n}}}{{2n}}$$

where *ε*_GAM_ is the experimental GAM efficiency, *ε* is the bead efficiency of in silico GAM and *n* is the number of beads composing a bin in the polymer structures. For example, we found that the value *ε* = 0.5 employed in Fig. 5 corresponds to *ε*_GAM_ = 0.97, which is very close to the experimental efficiency of 0.94 estimated for the published GAM dataset at 40-kb resolution^9^. We also tested, using numerical simulations, that the above approximate relation holds.

In the in silico experiments, the efficiency value used can strongly affect the quality of the contact maps (Supplementary Fig. 11), which worsens when efficiency is reduced. Such impact is generally compensated by the use of a large number of cells. Indeed, bulk contact maps, even at very low efficiency values, well correlate with those obtained at efficiency = 1. For instance, the Spearman correlation between bulk contact maps at efficiency = 1 and efficiency = 0.025 is *r*_s_ = 0.96 for Hi-C, *r*_s_ = 0.97 for SPRITE and *r*_s_ = 0.99 for GAM. Hence, for large numbers of cells, contact matrices are overall independent of efficiency, albeit that certain effects remain, linked to the specificity of the protocols of the experimental technologies.

### Analyses of in silico single-cell data

In silico Hi-C, SPRITE and GAM single-cell contact matrices were obtained from a single simulated cell—that is, a pair of independent polymer structures (Fig. 4c). Single-cell distance matrices were computed as described in the section In silico distance matrices. For a given simulated cell, we calculated Pearson, Spearman and HiCRep correlation coefficients between contact and distance maps and between the latter and the bulk distance matrix. This was repeated for 250 in silico single cells and the average correlation was extracted (Supplementary Table 1). The distributions of Spearman correlation are reported in Fig. 4d for contact maps at efficiency = 1. In the calculation of rank correlations (Spearman) we considered the dissimilarity of the data structure of SPRITE and of Hi-C and GAM in silico single-cell data, as the latter have only integer entries. Such a difference was taken into account by normalization and discretization of the entries of in silico SPRITE matrices in integers.

### Analysis of noise-to-signal ratio

The noise-to-signal ratio of the entry *a*_*ij*_ of a contact matrix, where *i* and *j* are bin indices, is defined as the ratio between standard deviation *σ*_*ij*_ and the mean *μ*_*ij*_ of that entry, across replicates. To estimate two such quantities, we first fixed the number of cells *N* and the efficiency *ε*. We then performed an in silico version of Hi-C, SPRITE and GAM 50 times each (when *N* = 1, we ran 100,000 times to overcome low sampling), and obtained a set of replicate contact matrices whereby we could extract mean and standard deviations for each entry, *a*_*ij*_. This procedure was applied for *N* and *ε* ranging from, respectively, 1–50,000 and 0.05–0.90. In particular, in Fig. 6 and Supplementary Figs. 8e–g, 9e–g, 10e–g and 14e–g we report the noise-to-signal ratio under different conditions.

First, we fixed the number of cells (*N* = 50,000) and efficiency (*ε* = 0.5) and studied variation in the noise-to-signal ratio with genomic distance (Fig. 6a and Supplementary Figs. 8, 9, 10 and 14e). To do that, we averaged over those entries corresponding to a fixed genomic distance, *d*—that is, considering all entries with *i* and *j* satisfying *d* = |*i* – *j*| × res, where res is resolution (40 kb). Analogously (Fig. 6b and Supplementary Figs. 8, 9, 10 and 14f), we fixed the genomic distance (*d* = 1 Mb, approximately the TAD length scale) and efficiency (*ε* = 0.5) and varied the number of cells. Noise-to-signal ratio was computed as average over those entries corresponding to *d* = |*i* – *j*| × res = 1 Mb, as before. Finally (Fig. 6c and Supplementary Figs. 8, 9, 10 and 14g) we fixed genomic length (1 Mb) and the number of cells (*N* = 50,000), and varied efficiency in the range 0.05–0.90.

### Heuristic criterion for assessment of similarity between replicates

Here we describe the criterion employed to assess the minimal number of cells (*M*) required for an in silico experiment (Hi-C, GAM or SPRITE) to provide reproducible contact maps—that is, contact matrices sufficiently similar across replicate experiments (Fig. 5d–f and Supplementary Figs. 8b–d, 9b–d, 10b–d, 14b–d and 15d). Given a simulated experiment with fixed efficiency *ε* and cell number *N*, we repeat it *k* independent times and correspondingly produce *k* contact matrices. The pool of *N* in silico cells employed each time is randomly chosen. We then compute the average Pearson correlation, *r* between all possible pairs of the contact matrices set. In general, as *N* increases so does *r*. If *r* equals *r*_t_ = 0.90, the corresponding number of cells employed, *M*, is taken as the minimum required for reproducibility at efficiency *ε*. To estimate *M* for a given efficiency, we employed a bisection search algorithm with the parameter *k* set to 15. Although our criterion is heuristic, our estimates of *M* for various efficiencies (Fig. 5d–f) are statistically robust and, importantly, they are consistent with the CLT (following section). We tested our approach with other, similar measures including Spearman and HiCRep correlation coefficients, and found similar results in the *Sox9* locus case study at efficiency = 0.1 (Supplementary Fig. 7).

### Consistency of the heuristic criterion with the CLT

The definition of the number of cells required for replicate similarity, *M*, based on their correlation value (Main Text), is fully consistent with a definition grounded on the CLT. In brief, consider the average value, *μ*, and the standard deviation, *σ*, of a given entry of a contact map in an experiment with *N* cells at a given efficiency. CLT dictates that the noise-to-signal squared ratio, *σ*^2^/*μ*^2^, scales as 1/*N*. Accordingly, from the CLT, the minimal number of cells, *L*, required to reduce the noise-to-signal squared ratio lower than a given threshold *δ* is *L* = *Aδ*^−2^*σ*^2^/*μ*^2^, where *A* is a constant. We checked that both *M* and the average value of *L* are linearly proportional to each other—that is, *M* is inversely proportional to the squared signal-to-noise ratio averaged over all entries of a single-cell contact map *ρ* (Supplementary Fig. 12). With analogous statistical arguments (following section), the approximate inverse squared power law relation between *M* and efficiency (Fig. 5f) can be explained.

Here, we discuss the above results in detail and show how the heuristic criterion used to define *M* (Main Text) is consistent with a simple argument based on the CLT: let the *b* × *b* matrix

$$\left( {\begin{array}{*{20}{c}} {a_{11}} & \cdots & {a_{1b}} \\ \vdots & \ddots & \vdots \\ {a_{b1}} & \cdots & {a_{bb}} \end{array}} \right)$$

be the output of a simulated single-cell experiment (for example, Hi-C, SPRITE or GAM). We suppose that each entry of the matrix is a random variable with a probability density function (pdf) *f*_*ij*_. This pdf in general depends parametrically on the global efficiency of the simulated experiment (*ε*). Thus, the following relation holds:

$$P\left\{ {a_{ij} \in \left[ {a,a + {\mathrm{d}}a} \right]} \right\} = f_{ij}\left( {a;\varepsilon } \right){\mathrm{d}}a,$$

where the first term is the probability of *a*_*ij*_ belonging to the interval [*a*,*a* + d*a*]. By definition, mean and variance for the entry (*i*,*j*) are

$$\mu _{ij}\left( \varepsilon \right) = {\int} {{\mathrm{d}}af_{ij}\left( {a;\varepsilon } \right)a} ,\quad \sigma _{ij}^2\left( \varepsilon \right) = {\int} {{\mathrm{d}}af_{ij}(a;\varepsilon )\left( {a - \mu _{ij}\left( \varepsilon \right)} \right)^2} .$$

If an in silico experiment with *N* cells at efficiency *ε* is performed, the output is the sum of *N* independent single-cell matrices:

$$\left( {\begin{array}{*{20}{c}} {A_{11} = \mathop {\sum }\limits_{m = 1}^n a_{11}^m} & \cdots & {A_{1b} = \mathop {\sum }\limits_{m = 1}^n a_{1b}^m} \\ \vdots & \ddots & \vdots \\ {A_{b1} = \mathop {\sum }\limits_{m = 1}^n a_{b1}^m} & \cdots & {A_{bb} = \mathop {\sum }\limits_{m = 1}^n a_{bb}^m} \end{array}} \right),$$

where *m* runs over the number of cells. We make the reasonable assumption that the $a_{ij}^m$ random variables, for fixed (*i,j*), are identically distributed. Thus, each entry *A*_*ij*_, if *N* is large enough, is a normally distributed random variable according to the CLT. Mean and variance are

$$M_{ij}\left( \varepsilon \right) = n\mu _{ij}\left( \varepsilon \right),\;{{S}}_{ij}^2\left( \varepsilon \right) = n\sigma _{ij}^2\left( \varepsilon \right).$$

Let us now suppose *N* is large enough so that the CLT holds. Then, for the entry *A*_*ij*_, we can impose a noise-to-signal ratio lower than a fixed value *δ*:

$$\frac{{{{S}}_{ij}(\varepsilon )}}{{M_{ij}(\varepsilon )}} \le \delta \to \frac{1}{{\sqrt n }}\frac{{\sigma _{ij}\left( \varepsilon \right)}}{{\mu _{ij}\left( \varepsilon \right)}} \le \delta \to n \ge \frac{1}{{\delta ^2}}\frac{{\sigma _{ij}^2\left( \varepsilon \right)}}{{\mu _{ij}^2\left( \varepsilon \right)}} \equiv L_{ij}(\varepsilon ).$$

Hence, in a simulated experiment with *N* cells, *N* needs to be higher than *L*_*ij*_(*ε*) for *A*_*ij*_ to have a noise-to-signal lower than *δ*. Consequently, we can define the minimum number of cells granting a stable entry *A*_*ij*_ as

$$L_{ij}\left( \varepsilon \right) = \delta ^{ - 2}\frac{{\sigma _{ij}^2\left( \varepsilon \right)}}{{\mu _{ij}^2\left( \varepsilon \right)}}.$$

To relate this quantity to the heuristic definition of minimal cell number, we need to generalize for all matrix entries. So, we average the noise-to-signal ratio over the entries (since contact matrices are symmetric, we can consider only the upper triangular submatrix):

$$\langle \frac{{\sigma ^2}}{{\mu ^2}} \rangle (\varepsilon ) = \frac{2}{{b(b - 1)}}\mathop {\sum }\limits_{j > i} \frac{{\sigma _{ij}^2\left( \varepsilon \right)}}{{\mu _{ij}^2\left( \varepsilon \right)}},$$

where *b*(*b* – 1)/2 is the number of upper triangular entries. This expression is dominated by single-cell entries with large noise and small signal. However, we want the definition of minimum number of cells to be controlled by high-signal and low-noise entries, which are more interesting. Therefore, it is more appropriate to average over the squared signal-to-noise ratio. By simply rewriting the definition of *L*_*ij*_ as

$$L_{ij}\left( \varepsilon \right) = \delta ^{ - 2}\left( {\frac{{\mu _{ij}^2(\varepsilon )}}{{\sigma _{ij}^2\left( \varepsilon \right)}}} \right)^{ - 1},$$

we can assume that the minimal cell number *L*(*ε*) yielding reproducibility for the whole *N*-cell experiment at efficiency *ε* is given by

$$L\left( \varepsilon \right) = \delta ^{ - 2}\rho ^{ - 1}\left( \varepsilon \right),$$

$$\rho \left( \varepsilon \right) \equiv \langle \frac{{\mu ^2}}{{\sigma ^2}} \rangle \left( \varepsilon \right) = \frac{2}{{b\left( {b - 1} \right)}}\mathop {\sum }\limits_{j > i} \frac{{\mu _{ij}^2\left( \varepsilon \right)}}{{\sigma _{ij}^2\left( \varepsilon \right)}}.$$

So, given the minimal cell numbers, *M*—found by our heuristic criterion (previous section) for different efficiencies—we tested whether these are consistent with the CLT-based definition by checking whether they are proportional to *ρ*^−1^(*ε*). In Supplementary Fig. 12 we explicitly show that this relation is well verified in our simulations.

### Statistical relation between *M* and *ε*

Based on the results in the previous section, we can derive the relation between *M* and *ε* (Fig. 5f and Supplementary Figs. 8–10 and 14d). Since *M* and *L*(*ε*) are proportional (see previous section), we need to derive only the dependence of *L*(*ε*) on *ε*.

Let us suppose that the output matrix of an in silico single-cell experiment is a Bernoullian variable—that is, it has only binary entries—for example, 0 or 1. This is not exactly the case for Hi-C and SPRITE, yet we will show that the following considerations hold approximately true. For a simulated single-cell experiment with efficiency *ε*, we define the probability for the entry *a*_*ij*_ to be 1 as *p*_*ij*_(*ε*) and have $\mu _{ij}(\varepsilon ) = p_{ij}(\varepsilon )$ and

$$\sigma _{ij}^2\left( \varepsilon \right) = p_{ij}\left( \varepsilon \right)\left( {1 - p_{ij}\left( \varepsilon \right)} \right),$$

where *μ*_*ij*_(*ε*) and ${\mu_{ij}}\left( \varepsilon \right) = p_{ij}\left( \varepsilon \right)$ indicate mean and variance, respectively. Hence, following the notation of the previous section, we can rewrite *ρ*(*ε*) in this particular case as

$$\rho \left( \varepsilon \right) = \frac{2}{{b(b - 1)}}\mathop {\sum }\limits_{j > i} \frac{{p_{ij}(\varepsilon )}}{{1 - p_{ij}(\varepsilon )}}.$$

To find the relation between *L*(*ε*) and *ε* we need to write *ρ*(*ε*) as function of *ε*—that is, to exhibit the dependence of *p*_*ij*_(*ε*) on *ε*. In complete generality, it holds:

$$p_{ij}\left( \varepsilon \right) = P\left\{ {a_{ij} = 1} \right\} = P\left\{ {i\;{\mathrm{detected}}} \right\}P\left\{ {j\;{\mathrm{detected}}} \right\}P\left\{ {i,j\;{\mathrm{in}}\;{\mathrm{contact}}{\mathrm{|}}i,j\;{\mathrm{detected}}} \right\},$$

where $\sigma _{ij}^2\left( \varepsilon \right)$ ($P\left\{ {j}\,{{\mathrm{detected}}} \right\}$) is the probability of bin *i* (*j*) being detected and $P\left\{ {i,j\;{\mathrm{in}\;{\mathrm{contact}}}{\mathrm{|}}{i,j}\;{\mathrm{detected}}} \right\}$ is the conditioned probability that bins *i* and *j* are in contact given that they have been detected. We now assume that $P\left\{ {i}\,{{\mathrm{detected}}} \right\} = P\left\{ {j}\,{{\mathrm{detected}}} \right\} = \varepsilon$. This is a simplifying assumption, because a bin is actually composed of multiple beads. We have:

$$p_{ij}\left( \varepsilon \right) = \varepsilon ^2c_{ij},$$

where, for sake of simplicity, we have indicated the conditioned probability with *c*_*ij*_.

So we can write *ρ*(*ε*) as function of *ε*:

$$\rho \left( \varepsilon \right) = \frac{2}{{b(b - 1)}}\mathop {\sum }\limits_{j > i} \frac{{\varepsilon ^2c_{ij}}}{{1 - \varepsilon ^2c_{ij}}}.$$

For small *ε*, we can approximate the denominator as 1 and have

$$\rho (\varepsilon )_{\varepsilon \ll 1} = \varepsilon ^2\frac{2}{{b(b - 1)}}\mathop {\sum }\limits_{j > i} c_{ij} = \varepsilon ^2c.$$

Thus, the Bernoulli approximation leads to a predicted exponent of −2 between *L*(*ε*) and *ε* for small values of *ε*:

$$L\left( \varepsilon \right) = \delta ^{ - 2}\rho ^{ - 1}\left( \varepsilon \right) \approx \delta ^{ - 2}\varepsilon ^{ - 2}.$$

These predictions are satisfactorily confirmed in Fig. 5f and Supplementary Figs. 8–10 and 14d). The fact that Hi-C and SPRITE entries are not binary would affect the functional form of *ρ*(*ε*), adding correction terms to the *ε*^2^ dependency. Nevertheless, for small efficiencies, *ε*^2^ is still expected to be the most relevant term and the approximations chosen still hold.

### Analysis of single-cell experimental data

We checked how our estimates of the number of cells required for replicate similarity, *M*, compare against available experimental investigations, and we verified that the correlation values between in silico replicates are comparable to those found in experiments.

We considered published Hi-C data^55^ from the mouse CD4 T_H_1 cell line. Available data include bulk Hi-C, ten single-cell Hi-C and pooled Hi-C from 60 single-cell experiments. For all of these, we extracted 40-kb Hi-C maps for the *Sox9* locus (chr11:109–115 Mb, mm9) by totaling contact counts belonging to the same 40-kb bin pairs. We calculated the mean Spearman correlation between all possible independent pairs of single-cell Hi-C maps, obtaining *r*_s_ = 0.01 (Supplementary Fig. 6a). Next, we computed the Spearman correlation between bulk Hi-C and 60-cell maps, obtaining *r*_s_ = 0.33 (Supplementary Fig. 6b and Main Text).

Then, from the Hi-C-derived 3D structures of the *Sox9* locus in mESC, we generated an in silico bulk Hi-C map, a 60-cell map and ten single-cell maps produced at efficiency = 0.025. This is the upper bound efficiency declared for the Hi-C data above^55^. We calculated the average Spearman correlation between all independent pairs of in silico single-cell maps, obtaining *r*_s_ = 0.01, which is numerically equal to the mean correlation found between single-cell Hi-C maps from the CD4 T_H_1 cell line (Supplementary Fig. 6a). Finally, Spearman correlation was computed between the bulk and 60-cell Hi-C maps, finding *r*_s_ = 0.27, which is not far from the correlation *r*_s _= 0.33 obtained in the experimental analog presented above (Supplementary Fig. 6b).

Additionally, to verify that our estimates of *M* are consistent with available experimental results, we considered the data from a recent Low-C experiment (a Hi-C method for low input material) on mESC^60^ where, in the case of a locus of width 10 Mb, a sample of 1,000 cells was shown to be sufficiently large to produce contact maps highly similar to the bulk map (Pearson correlation *r* = 0.95). That estimate of the minimal number of cells needed to approach the bulk limit is consistent with the value of *M* = 650 for in silico Hi-C at efficiency = 0.05, as reported in the Main Text.

### Structural comparison of single-molecule 3D conformations from model and multiplex FISH microscopy

To check whether the SBS 3D structures are a bona fide representation of chromatin conformations in single cells, we considered the super-resolution microscopy data of the HCT116 locus available in the literature^22^ at 30 kb. The model 3D structures^28^ were compared to the HCT116 experimental configurations via the RMSD criterion^28,54^, which comprises rotation of two centered 3D structures until their coordinate difference is minimal, where that difference is computed by the RMSD of corresponding site coordinates. Thus, for every model configuration, the best-matching experimental structure is that yielding the lowest RMSD. To prove their significance, the best RMSD values found for each model configuration were tested against a control distribution, given by RMSD between an ensemble of SAW polymer structures and their best-matching experimental conformations. To obtain a fair control, the SAW model structures were produced with the same number of particles and the same average gyration radius as the imaged conformations. The two distributions were found statistically different (two-sided Mann–Whitney *U*-test *P* = 0; Fig. 2b). Analogously, the distribution of RMSD between the imaging data and their best-matching SBS structures was considered and compared to that of RMSD between experimental and best-matching SAW structures. Again, the distributions were found statistically incompatible (two-sided Mann–Whitney *U*-test *P* = 0; Fig. 2c). Finally, as in a previously published paper^28^, we compared the distribution of RMSD between the experimental structures and their best-matching SBS model to that of RMSD between best-matching pairs of experimental structures. The distributions were found to be statistically indistinguishable, with two-sided Mann–Whitney *U*-test *P* = 0.15.

### Correlations between distance maps from 3D conformations of model and multiplex FISH microscopy

In the case of the human HCT116 locus, we considered the single-molecule distance maps of the imaged 3D structures^22^ and of the SBS model configurations^28^ and computed the correlations between all pairs (exp–SBS distribution of correlations; Supplementary Fig. 5d). Analogously, we considered the single-molecule distance maps of the SAW 3D structures (see previous paragraph) and calculated all-against-all correlations with the experimental single-molecule distance matrices (exp–SAW distribution). Finally, correlations between all possible pairs of experimental single-molecule distance maps were extracted (exp–exp distribution). Specifically, in all three cases we computed the Pearson genomic-distance-corrected correlations^27,28^, *r'* (Supplementary Fig. 5d): for every pair of considered matrices, we first subtracted from each of their diagonals their mean values, to remove the average effect of increasing distance with increasing genomic separation; then, the Pearson correlation was calculated. The exp–SBS and exp–exp distributions are statistically indistinguishable (two-sided Mann–Whitney *U*-test *P* = 0.19 (ref. ^28^); average values *r'* = 0.21 and *r'* = 0.27, respectively). Conversely, the exp–SAW distribution is statistically different from both (two-sided Mann–Whitney *U*-test *P* = 0; average *r'* = 0.00). We also computed the *r'* correlation between the mean experimental distance matrix and the mean distance matrices from the SBS and SAW model structures (Supplementary Fig. 5a–c) where we found, respectively, *r'* = 0.84 and *r'* = 0.32.

### Statistical significance of HiCRep scores between in silico and experimental contact maps

Albeit that HiCRep has been devised to compare pairs of Hi-C matrices, we used it also for pairs of GAM and SPRITE data to return a comprehensive view of similarity measures, beyond Spearman and Pearson correlations. We verified that the HiCRep correlations (scc) between experimental and in silico GAM and SPRITE contact maps are statistically significantly high. In the case of the *Sox9* locus (Fig. 1b and Supplementary Table 1a), we considered a null model where we computed scc between 100 pairs of randomized experimental and in silico contact maps, for Hi-C, SPRITE and GAM. Randomization is performed by bootstrapping contact frequencies at each genomic distance. We found that the measured scc correlations between model and experimental maps are higher than the 90th percentiles of the control distributions for all three technologies (Supplementary Fig. 3). For all other loci, analogous results were obtained.

### Reporting Summary

Further information on research design is available in the Nature Research Reporting Summary linked to this article.

## Online content

Any methods, additional references, Nature Research reporting summaries, source data, extended data, supplementary information, acknowledgements, peer review information; details of author contributions and competing interests; and statements of data and code availability are available at 10.1038/s41592-021-01135-1.

## Supplementary information

Supplementary Information Supplementary Table 1 and Figs. 1–16.

Reporting Summary

## Data Availability

Published Hi-C, SPRITE, GAM and microscopy data used for analysis are available at the referenced papers. The new GAM data from the F123 cell line are available on the 4D Nucleome data portal under accession no. 4DNFIFBSQ1EO.

## Code availability

The codes used in our work are based on standard, publicly available software packages, as detailed in Methods. Molecular dynamics simulations use LAMMPS, v.30july2016. Analyses and plots were produced with the Anaconda package v.4.7.12. HiCRep correlations were computed with R v.3.5.1. 3D structure visualizations were produced with POV-Ray, v.3.7. The algorithms for simulation of Hi-C, SPRITE, GAM and SLICE in silico use standard routines, such as DBSCAN, and are described in full detail in Methods. The Hi-C, SPRITE and GAM algorithms are available at https://github.com/fmusella/In-silico_Hi-C_GAM_SPRITE.
