# Publisher Correction: Comparison of the Hi-C, GAM and SPRITE methods using polymer models of chromatin

**DOI:** 10.1038/s41592-021-01251-y

**Published:** 2021-09-03

**Authors:** Luca Fiorillo, Francesco Musella, Mattia Conte, Rieke Kempfer, Andrea M. Chiariello, Simona Bianco, Alexander Kukalev, Ibai Irastorza-Azcarate, Andrea Esposito, Alex Abraham, Antonella Prisco, Ana Pombo, Mario Nicodemi

**Affiliations:** 1 Dipartimento di Fisica, Università di Napoli Federico II and INFN Napoli, Complesso Universitario di Monte Sant’Angelo, Naples, Italy; 2 Berlin Institute for Medical Systems Biology, Max-Delbrück Centre for Molecular Medicine, Berlin, Germany; 3 Humboldt-Universität zu Berlin, Berlin, Germany; 4 CNR-IGB, Naples, Italy; 5 Berlin Institute of Health, Berlin, Germany

**Keywords:** Computational models, Computational biophysics, Biological techniques

Correction to: *Nature Methods* 10.1038/s41592-021-01135-1, published online 7 May 2021.

This paper was originally published under standard Springer Nature license (© The Author(s), under exclusive licence to Springer Nature America, Inc.). It is now available as an Open Access paper under a Creative Commons Attribution 4.0 International license, © The Author(s). The error has been corrected in the HTML and PDF versions of the article.

