# Supplementary material for: Comparison of the Hi-C, GAM and SPRITE methods using polymer models of chromatin
Source: Nat Methods. 2021 May 7;18(5):482–90. doi: 10.1038/s41592-021-01135-1 (PMC8416658; doi:10.1038/s41592-021-01135-1)
Supplement: Supplementary file 1 — Supplementary Table 1 and Figs. 1–16. [file 41592_2021_1135_MOESM1_ESM.pdf]

nature portfolio

https://doi.org/10.1038/s41592-021-01135-1

Supplementary information

Comparison of the Hi-C, GAM and SPRITE methods using polymer models of chromatin

In the format provided by the authors and unedited

Supplementary Information

for

Comparison of the Hi-C, GAM and SPRITE methods by use of polymer models of chromatin

Luca Fiorillo[1,#], Francesco Musella[1,#], Mattia Conte[1,#], Rieke Kempfer[2,3], Andrea M. Chiariello[1], Simona Bianco[1,2], Alexander Kukalev[2], Ibai Irastorza Azcarate[2], Andrea Esposito[1], Antonella Prisco[4], Alex Abraham[1], Ana Pombo[2,3,5], Mario Nicodemi[1,2,5,*]

[1] Dipartimento di Fisica, Università di Napoli *Federico II*, and INFN Napoli, Complesso Universitario di Monte Sant'Angelo, 80126 Naples, Italy.

[2] Berlin Institute for Medical Systems Biology, Max-Delbrück Centre (MDC) for Molecular Medicine, Berlin, Germany.

[3] Humboldt-Universität zu Berlin, 10117 Berlin, Germany

[4] CNR-IGB, via Pietro Castellino 111, Naples, Italy

[5] Berlin Institute of Health (BIH), Berlin, Germany.

[#] These authors contributed equally

* Lead contact: mario.nicodemi@na.infn.it

SUPPLEMENTARY TABLES

a)

	Hi-C		
Hi-C	0.83	0.83	0.80
	r	r_s	HiCRep

	SPRITE		
SPRITE	0.75	0.92	0.57
	r	r_s	HiCRep

	GAM		
GAM	0.80	0.79	0.40
	r	r_s	HiCRep

b) bulk

bulk	Distance		
Hi-C	-0.54	-0.89	-0.73
SPRITE	-0.72	-0.94	-0.52
GAM	-0.94	-0.99	-0.93
	r	r_s	HiCRep

c)

	Distance bulk		
Distance single-cell	0.87	0.88	0.55
	r	r_s	HiCRep

d) single cell

single cell	Distance		
Hi-C	-0.31	-0.37	-0.61
SPRITE	-0.37	-0.46	-0.57
GAM	-0.13	-0.15	-0.07
	r	r_s	HiCRep

e) single cell

single cell	Distance		
Hi-C	-0.06	-0.06	-0.22
SPRITE	-0.13	-0.16	-0.36
GAM	-0.13	-0.15	-0.08
	r	r_s	HiCRep

Supplementary Table 1. Pearson, Spearman and HiCRep correlations for the *Sox9* locus.

a) Pearson (r), Spearman (r_s) and HiCRep (scc) correlations between bulk Hi-C, SPRITE and GAM (from 1122 slices) experimental maps and the corresponding *in-silico* maps (see **Figure 1** and **Methods**), for the *Sox9* locus.

b) Pearson, Spearman and HiCRep correlations between the average distance map and the *in-silico* bulk Hi-C, SPRITE and GAM contact maps (see **Figure 3**) for the *Sox9* locus.

c) Pearson, Spearman and HiCRep mean correlations between *in-silico* single-cell distance maps and the average distance map for the *Sox9* locus (see **Figure 4a,b**).

d) Mean correlations between *in-silico* single-cell contact maps - at efficiency 1 - and the corresponding single-cell distance map (see **Figure 4c,d**).

e) Same as panel d), but here *in-silico* contact maps are generated with efficiencies similar to the experimental ones (0.05 for Hi-C and SPRITE and 0.5 for GAM; see **Main Text** and **Methods**). The reduction of efficiency worsens the similarity with the single-cell distance pattern.

SUPPLEMENTARY FIGURES

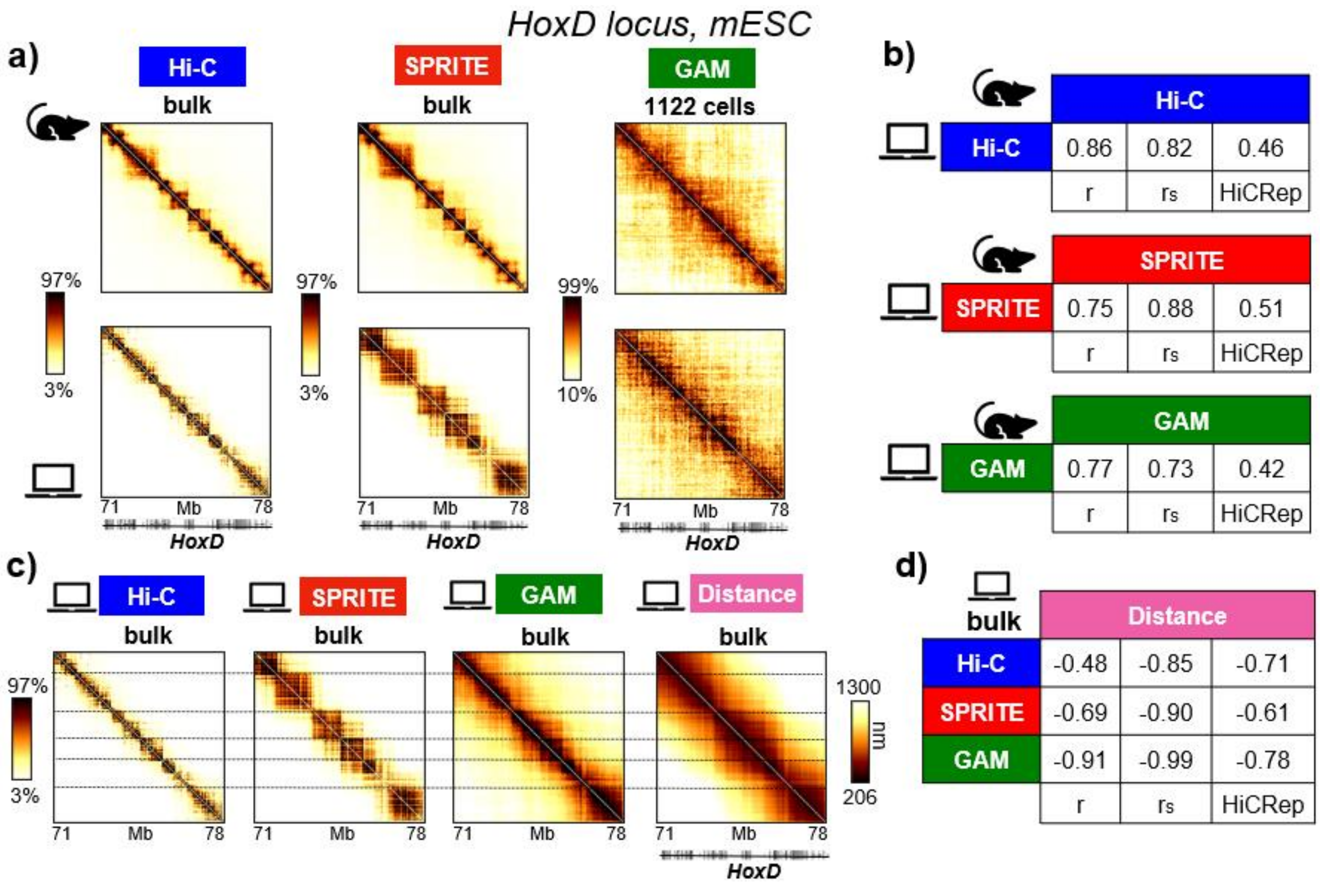

Supplementary Figure 1. ***In-silico*** **contact and distance data of the** ***HoxD*** **locus in mESC.**

a) In the case of the murine *HoxD* locus (chr2:71Mb-78Mb, mm9) in mESC, our 3D conformations[26] return *in-silico* contact maps (bottom) that match well with Hi-C[19], SPRITE[10] and GAM experimental data from mESC (top). GAM data are from the new dataset of 1122 nuclear slices (**Methods**). Correspondingly, *in-silico* Hi-C and SPRITE are bulk data, while *in-silico* GAM data are from 1122 *in-silico* cells (see **Methods**). Color scale indicates the percentiles of the maps.

b) Pearson, Spearman and HiCRep correlations between the *in silico* and experimental maps of panel a), for the three technologies.

c) *In-silico* bulk Hi-C, SPRITE and GAM maps of the *HoxD* locus return contact patterns that are compatible with the average distance pattern derived from the ensemble of single-molecule 3D conformations. The horizontal lines are drawn to mark the domain-like structure of the distance map.

d) Pearson, Spearman and HiCRep correlations are reported between the *in-silico* bulk contact maps and the average distance map. Correlations indicate a high degree of similarity, showing that the three technologies all faithfully capture the underlying conformations of the *HoxD* locus.

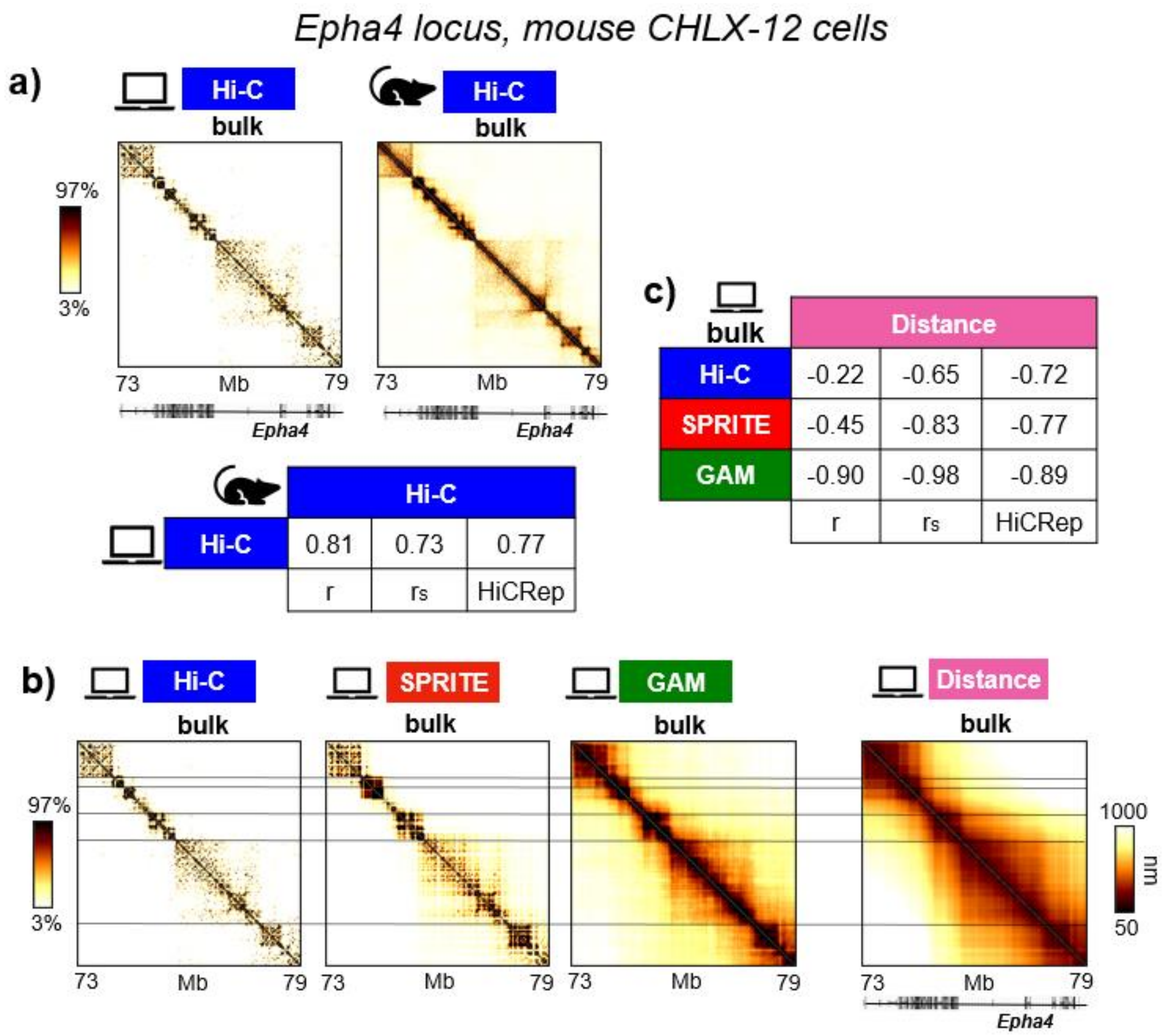

Supplementary Figure 2. *In-silico* contact and distance data of the *Epha4* locus in murine CHLX-12 cells.

a) In the model of the *Epha4* locus[27] (chr1:73-79Mb, mm9) in mouse CHLX-12 cells the *in-silico* bulk Hi-C map (left) is compared to the experimental map[4] (right) (color scale indicates the percentiles of the maps). In the table on the bottom, Pearson, Spearman and HiCRep correlations are reported, indicating good similarity between the two matrices.

b) The *in-silico* bulk contact maps are compared with the average distance pattern obtained from the ensemble of 3D conformations of the model of the locus. The horizontal lines are drawn to mark the domain-like structure of the distance map.

c) Pearson, Spearman and HiCRep correlations are reported between each bulk contact map and the average distance map, indicating overall a good degree of similarity for each of the technologies.

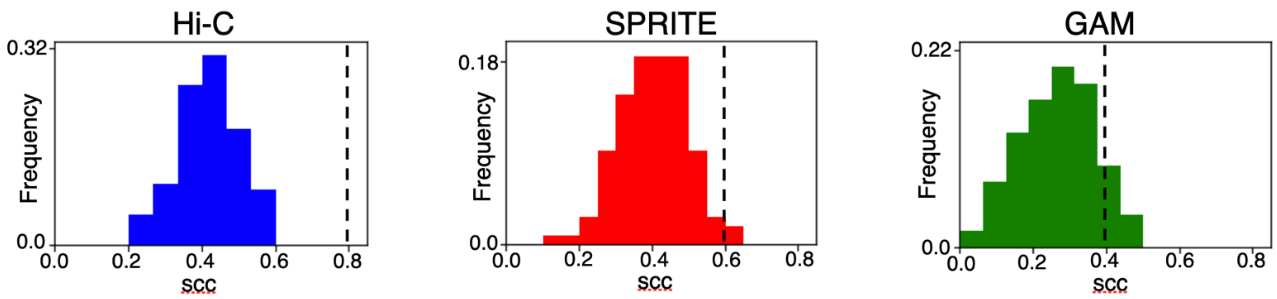

Supplementary Figure 3. HiCRep correlations between *in-silico* and experimental contact maps in the *Sox9* locus are statistically significantly high.

For the *Sox9* polymer model derived from Hi-C, the HiCRep correlations (ssc) between the bulk *in-silico* and experimental contact maps[9,10,19] (**Figure 1b**, **Supplementary Table 1a**) are compared against a random control distribution (**Methods**). On the left, the scc between the *in-silico* and experimental Hi-C matrices (dashed line) is above the 90th percentile of the control distribution (in blue), given by the scc between pairs of randomized *in-silico* and experimental Hi-C maps (**Methods**). That is true also for SPRITE (middle) and GAM (right), albeit HiCRep scores were originally designed to compare Hi-C data.

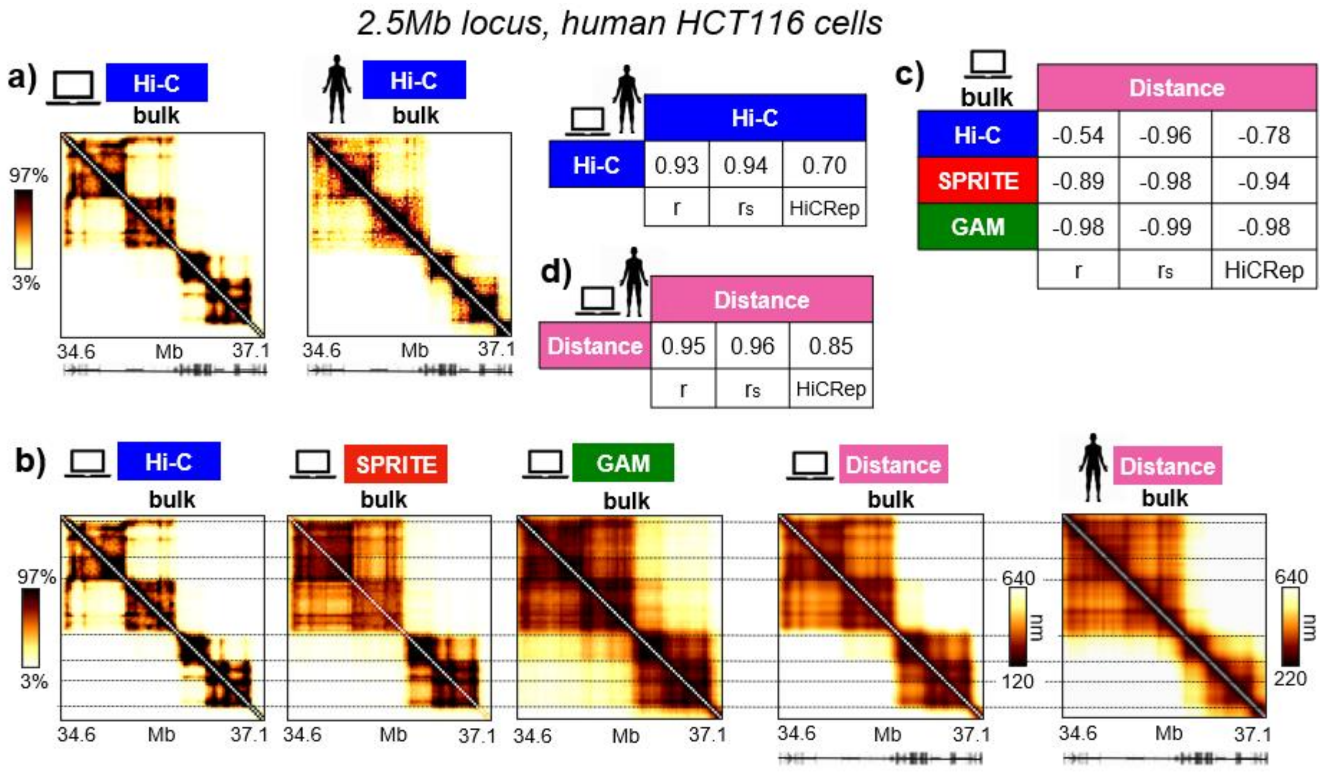

Supplementary Figure 4. ***In-silico*** **contact and distance data of the locus in human HCT116 cells.**

a) In the case of the locus from the human HCT116 cell line (chr21:36.4-37.1Mb), the *in-silico* Hi-C map derived from our 3D polymer configurations[28] is very similar to the Hi-C experimental matrix[53] (**Figure 2**). This is quantitatively expressed by the high correlation coefficients (Pearson, Spearman and HiCRep) reported in the table on the right. Color scale indicates the percentiles of the maps.

b) The *in-silico* bulk contact maps (first three matrices on the left) are shown together with the average distance matrix computed from the ensemble of 3D conformations. All three contact maps return overall faithfully the distance pattern (see also panel c)). The horizontal lines mark the domains detected. Additionally, consistent with the findings in panel a), the *in-silico* average distance map is very similar to that observed by super-resolution microscopy[22] (last matrix on the right)**.**

c) Pearson, Spearman and HiCRep correlations are listed between each *in-silico* bulk contact map and the *in-silico* distance matrix. Correlation coefficients are generally high for all three methods, indicating nice similarity with the distance pattern.

d) Pearson, Spearman and HiCRep correlations are reported between the *in-silico* and experimental average distance matrices. Correlation coefficients indicate great similarity between the two maps.

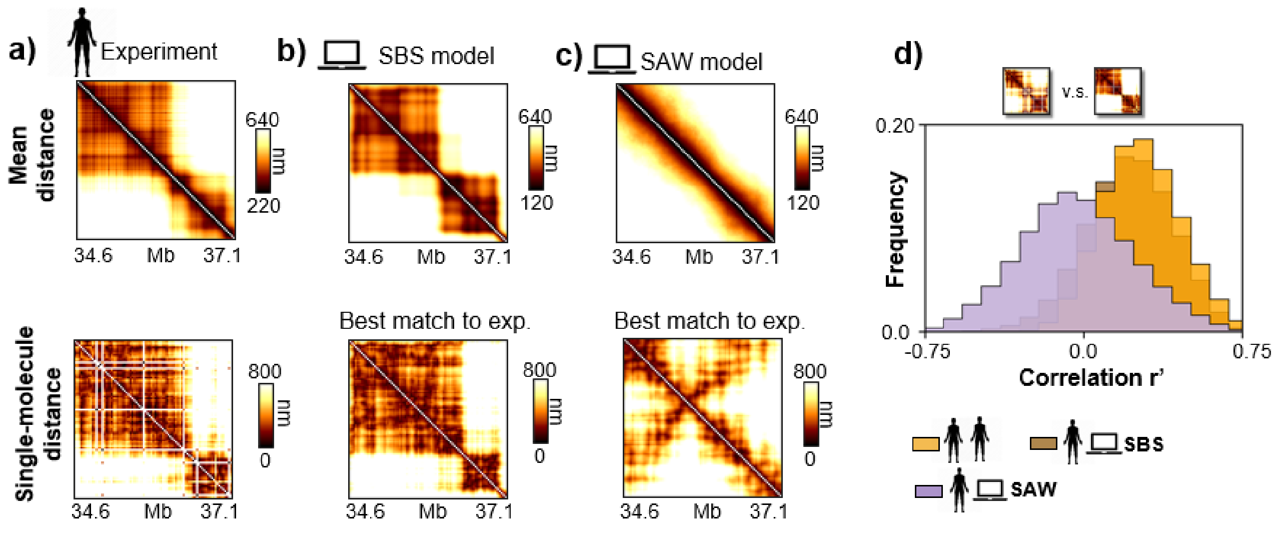

Supplementary Figure 5. Single-molecule distance maps of the SBS model of the human HCT116 locus significantly correlate with single-cell imaging data.

The mean distance matrix (top) and a typical example of single-molecule distance matrix (bottom) are shown from: a) imaging data of the human HCT116 locus[22]; b) the SBS model of the locus[28]; c) a control model made of self-avoiding random-walk (SAW) conformations (**Main Text** and **Methods**). The single-molecule conformations of the models in panel b) and c) are the best matching structures of the experimental one in panel a) according to the least RMSD criterion (**Figure 2, Methods**). The genomic-distance corrected Pearson correlation coefficient (r') is r'=0.84 between the experiment and SBS model mean distance matrices, and r'=0.32 between the experiment and SAW model ones. d) The distribution is shown of the r' values between pairs of experiment-experiment (orange), of experiment-SBS (brown) and of experiment-SAW (violet) single-molecule distance matrices. The experiment-experiment and experiment-SBS distributions are statistically indistinguishable (2-sided Mann-Whitney U test p-value = 0.19), but they are statistically different from the experiment-SAW distribution (2-sided Mann-Whitney U test p-value = 0).

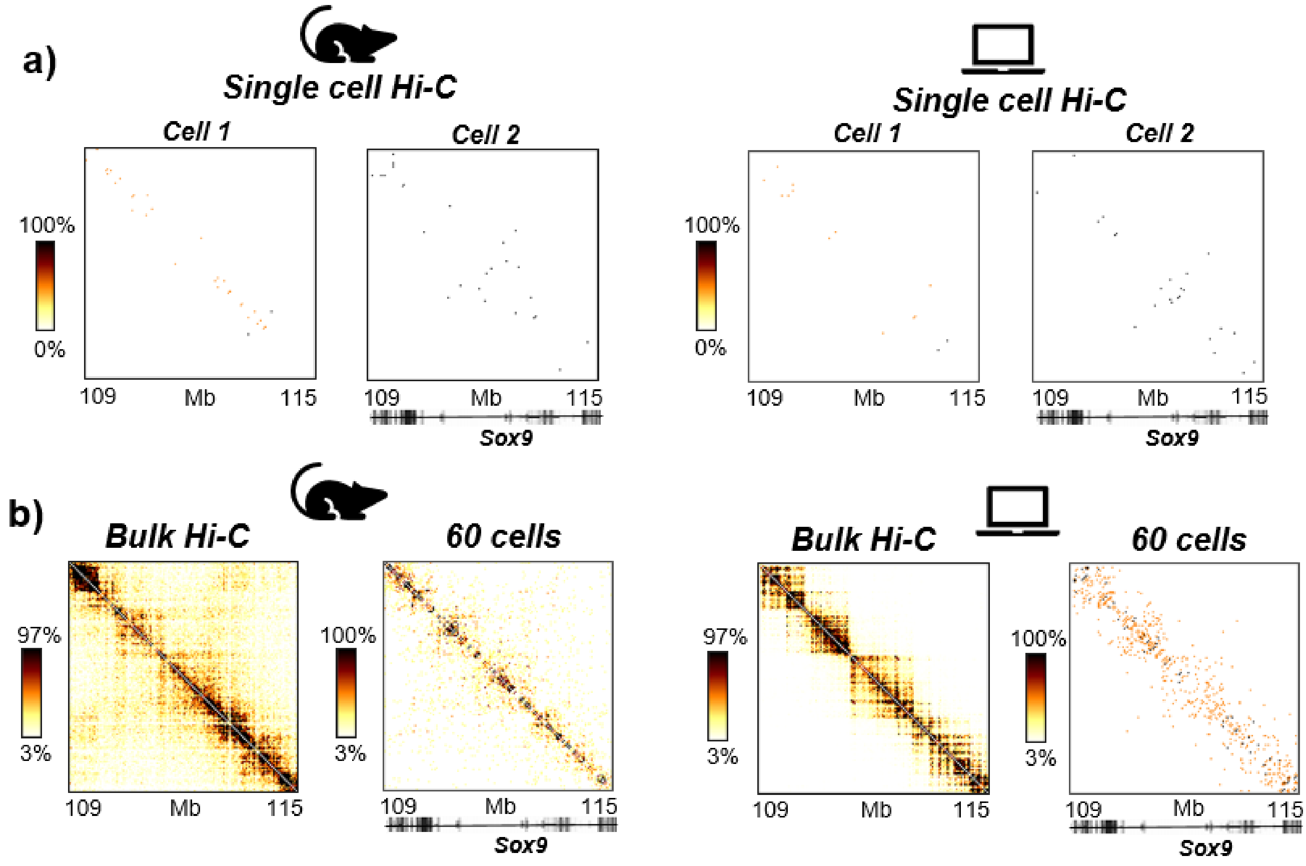

Supplementary Figure 6. Experimental and *in-silico* single-cell Hi-C data.

a) Left panel. Two examples of experimental single-cell Hi-C contact maps[55], for the *Sox9* locus in the mouse CD4 T_H1 cells. The mean Spearman correlation between all the available pairs of such single-cell *Sox9* maps[55] is r_S=0.01 (see also **Methods**). Right panel. Two examples of the *in-silico* single-cell Hi-C maps for the *Sox9* locus in mESC. Mean Spearman correlation is r_S=0.01, consistent to the experimental result (**Methods**). The efficiency is set to 0.025[55]. Color scale indicates the percentiles of the maps.

b) Left panel. In the same genomic region in CD4 T_H1 cells, the average experimental map resulting from 60 available single-cell contact data is compared against the bulk Hi-C map[55]: their Spearman correlation is r_S=0.33 (see **Methods**). Right panel. A similar calculation from the *in-silico* Hi-C maps in mESC returns a Spearman correlation between the bulk and the 60-cell map of r_s=0.27, close to the experimental value (**Methods**). Color scale indicates the percentiles of the maps.

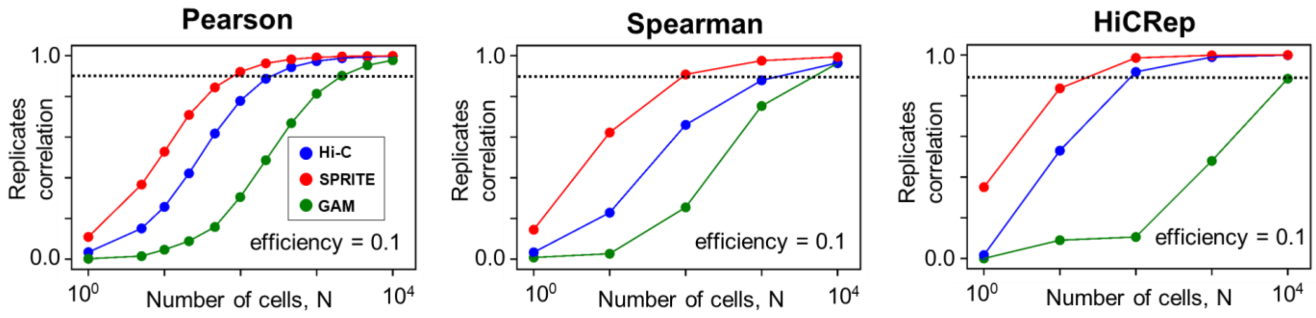

Supplementary Figure 7. Pearson, Spearman and HiCRep correlations between replicates in relation to the number of cells considered in the *in-silico* experiments.

The Pearson, Spearman and HiCRep correlations between replicate *in-silico* contact maps are shown for Hi-C, SPRITE and GAM at efficiency 0.1, in the case of the model of the *Sox9* locus. Dashed lines in each plot indicates the considered 0.9 threshold value (see **Figure 5c,d,e**).

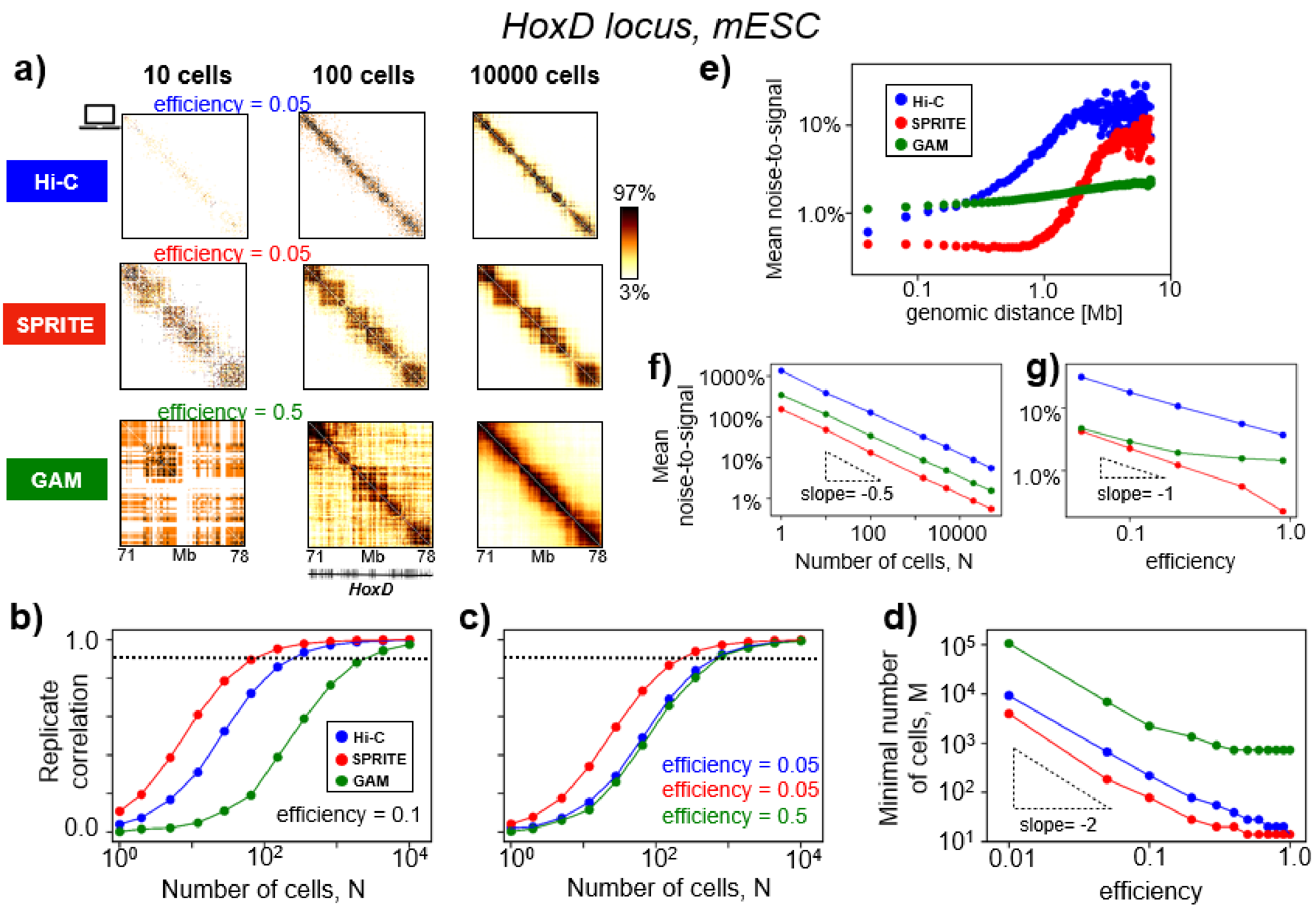

Supplementary Figure 8. Replicates reproducibility and noise level in the *HoxD* locus.

a) The *in-silico* Hi-C, SPRITE and GAM contact maps of the *HoxD* locus depend on the number of *in-silico* cells, N, considered in the experiment. Here it is shown the case where efficiencies similar to those found in real experiments are employed (0.05 for Hi-C and SPRITE, 0.5 for GAM; see **Figure 5**). In the bulk limit (large N) the effects of cell-to-cell variability are averaged out. Color scale indicates the percentiles of each map.

b) The Pearson correlation is shown between replicates as function of N at a given efficiency (0.1). The dashed line is the threshold correlation value r_t=0.9 (**Main Text** and **Methods**).

c) Results analogous to those in panel b) are shown for efficiencies similar to those of real experiments, as discussed in panel a).

d) The minimal number of cells, M, required for replicates reproducibility is shown against the efficiency for Hi-C, SPRITE and GAM. M increases as the efficiency is reduced and grows approximately as an inverse squared power law at small efficiencies, consistent with the Central Limit Theorem (**Methods**, **Supplementary Figures 11,12**). For a given efficiency, M is the smallest in SPRITE and the highest in GAM.

e) The mean noise-to-signal ratio $\langle \sigma/\mu \rangle$ (**Main Text** and **Methods**) of a contact map, for a given number of cells N and efficiency, depends on the considered genomic separation. In Hi-C and SPRITE, the noise-to-signal ratio drastically grows above 1Mb (the case shown is for N=50000 and efficiency=0.5).

f) For fixed genomic distance and efficiency (the case shown is for 1Mb and efficiency 0.5), the noise-to-signal ratio decreases with the number of cells as an inverse square root, as expected from the Central Limit Theorem.

g) For fixed genomic distance and number of cells (the case shown is for 1Mb and N=50000), the noise-to-signal ratio increases approximately as an inverse power law when the efficiency is reduced. All the above findings are fully consistent with those found for the *Sox9* locus (**Figures 5,6)** and all the other considered loci in murine and human cells.

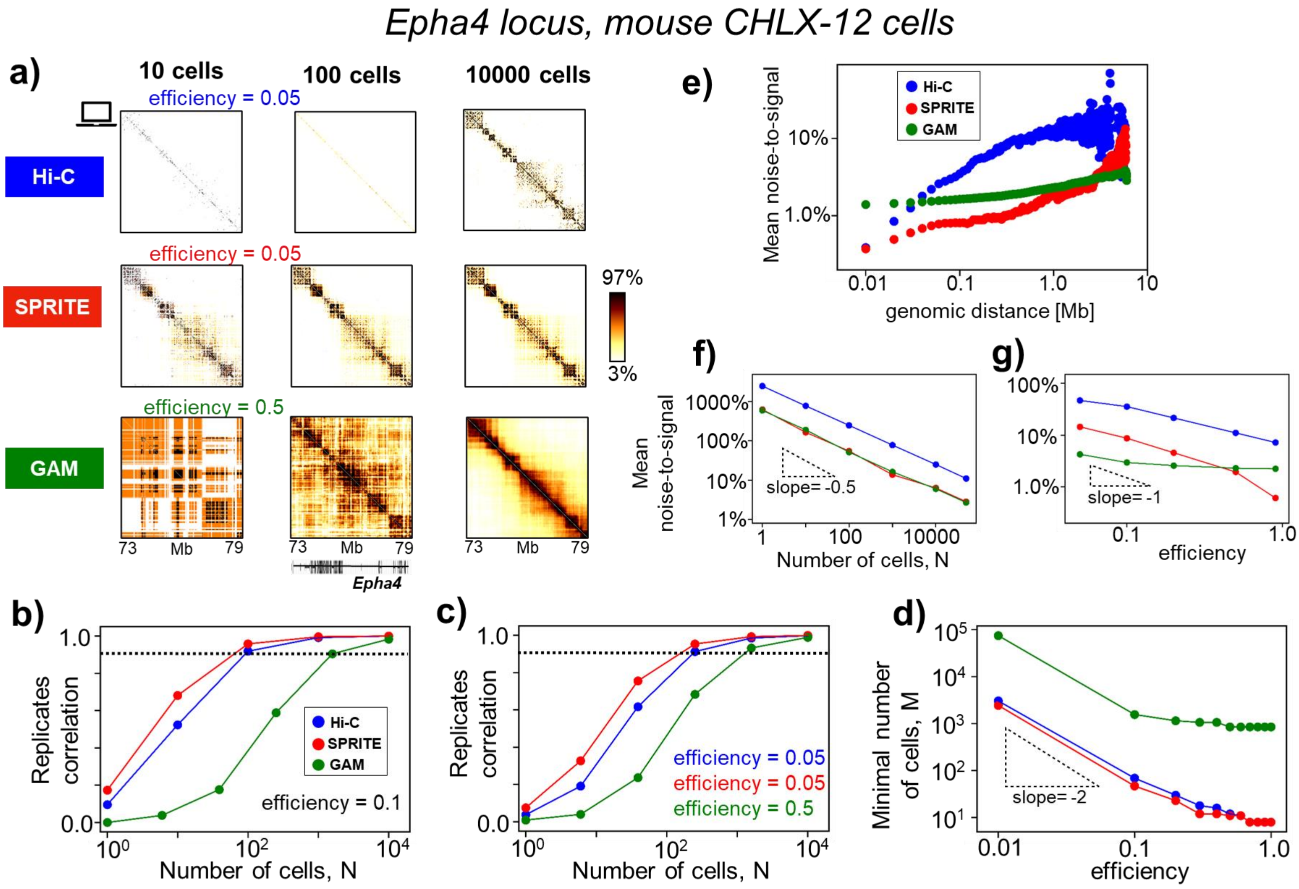

Supplementary Figure 9. Replicates reproducibility and noise level in the *Epha4* locus in murine CHLX-12 cells.

a) The *in-silico* Hi-C, SPRITE and GAM contact maps of the *Epha4* locus depend on the number of cells used in the experiment. Efficiencies analogous to those found in real experiments are employed (0.05 for Hi-C and SPRITE, 0.5 for GAM). Color scale indicates the percentiles of each map.

b) The Pearson correlation is shown between replicates as function of N at a given efficiency (0.1). The dashed line is the threshold correlation value r_t=0.9 (**Main Text** and **Methods**).

c) Similar results as panel b) are obtained when efficiencies similar to those of real experiments are employed.

d) The minimal number of cells, M, required for replicate reproducibility is shown against the efficiency for Hi-C, SPRITE and GAM. For small efficiencies, M grows approximately as an inverse squared power law when the efficiency is decreased, as expected from the Central Limit Theorem (**Methods**).

e) The average noise-to-signal ratio $\langle\sigma/\mu\rangle$ of a contact map, for given number of cells and efficiency, depends on the considered genomic distance (the case shown is for N=50000 and efficiency=0.5). For Hi-C and SPRITE, $\langle\sigma/\mu\rangle$ increases drastically above 1Mb.

f) For fixed genomic distance and efficiency (the case shown is for 1Mb and efficiency 0.5), the noise-to-signal ratio decreases with the number of cells as an inverse square root, as expected from the Central Limit Theorem.

g) For fixed genomic distance and number of cells (the case shown is for 1Mb and N=50000), the noise-to-signal ratio increases approximately as an inverse power law when the efficiency is reduced. All the above findings are fully consistent with those found for the *Sox9* locus (**Figures 5,6**) and all the other considered loci in murine and human cells.

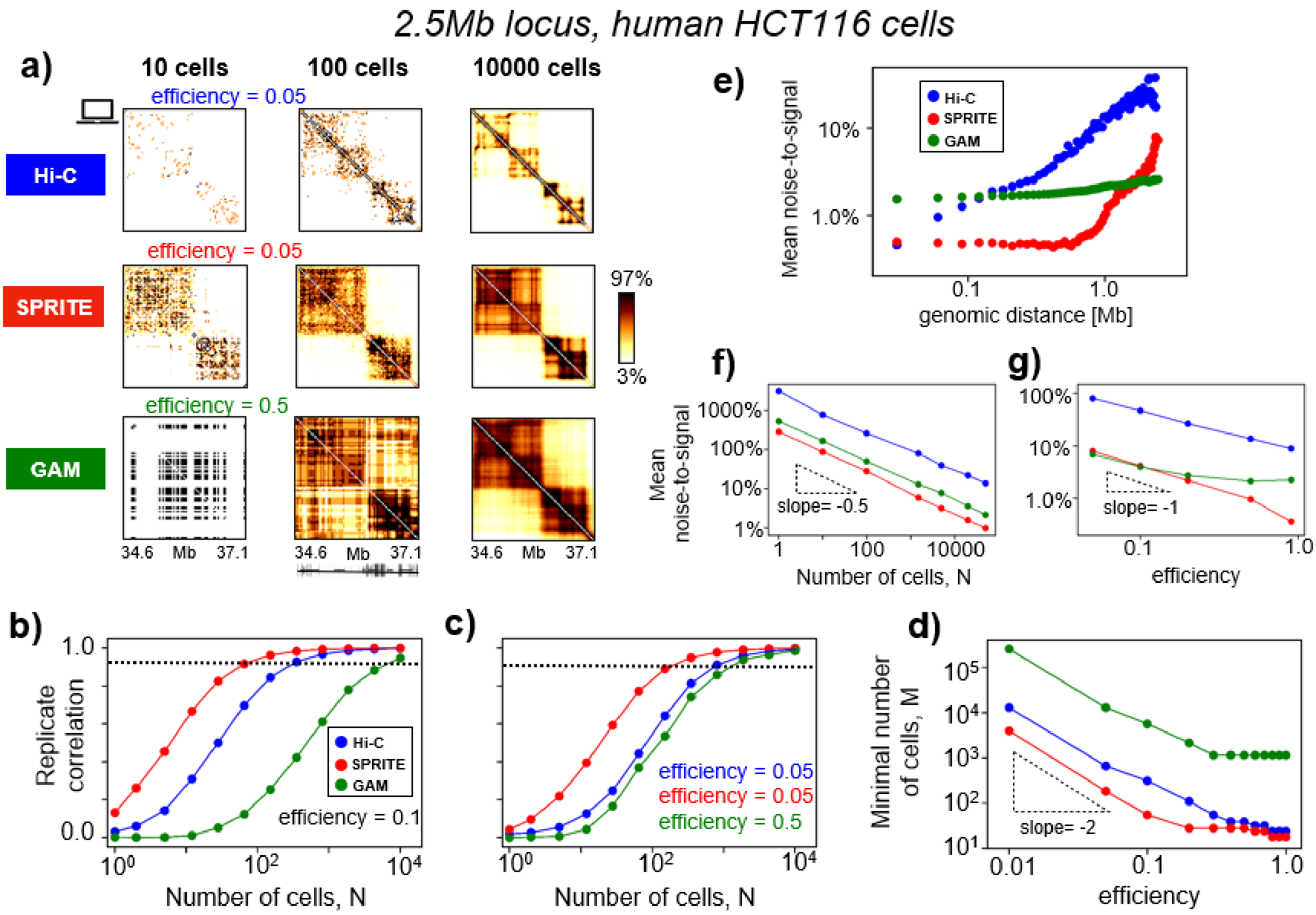

Supplementary Figure 10. Replicates reproducibility and noise level in the locus in human HCT116 cells.

a) The *in-silico* Hi-C, SPRITE and GAM contact maps of the HCT116 locus depend on the number of cells used in the experiment. Here, efficiencies analogous to those found in real experiments are used (0.05 for Hi-C and SPRITE, 0.5 for GAM). Color scale indicates the percentiles of each map.

b) The Pearson correlation is shown between replicates as function of N at a given efficiency (0.1). The dashed line is the threshold correlation value r_t=0.9 (**Main Text** and **Methods**).

c) Similar results as panel b) are obtained when efficiencies similar to those of real experiments are employed (as discussed in panel a)).

d) The minimal number of cells, M, required for replicates reproducibility is shown against the efficiency for Hi-C, SPRITE and GAM. For small efficiencies, M grows approximately as an inverse squared power law when efficiency is decreased, as expected from the Central Limit Theorem (**Methods**).

e) The average noise-to-signal ratio $\langle\sigma/\mu\rangle$ of a contact map, for given number of cells and efficiency, depends on the considered genomic distance (the case shown is for N=50000 and efficiency=0.5). For Hi-C and SPRITE, $\langle\sigma/\mu\rangle$ increases drastically above 1Mb.

f) For fixed genomic distance and efficiency (the case shown is for 1Mb and efficiency 0.5), the noise-to-signal ratio decreases with the number of cells as an inverse square root, as expected from the Central Limit Theorem.

g) For fixed genomic distance and number of cells (the case shown is for 1Mb and N=50000), the noise-to-signal ratio increases approximately as an inverse power law when the efficiency is reduced. All the above findings are fully consistent with those found for the *Sox9* locus (**Figures 5,6**) and all the other considered loci in murine and human cells.

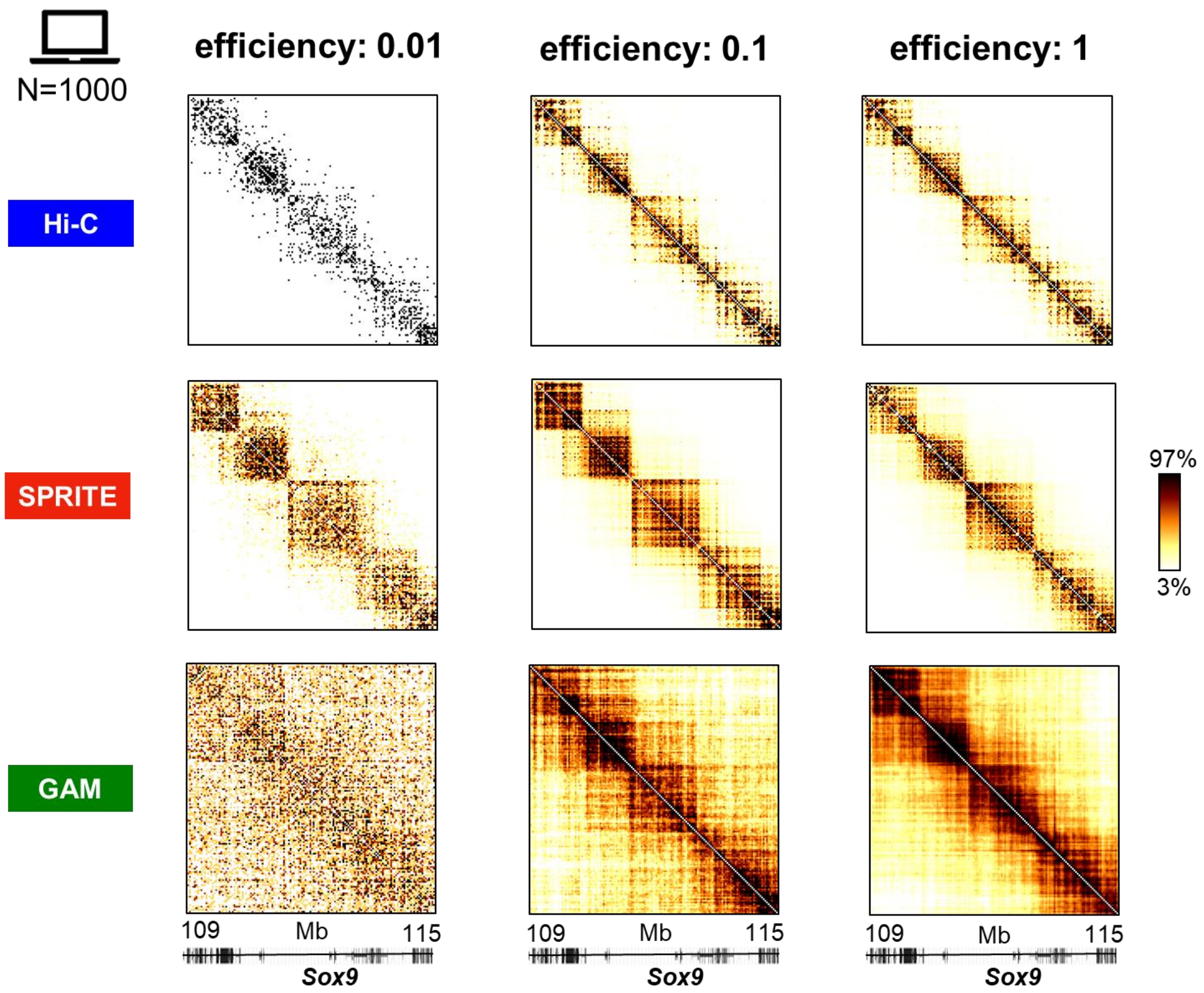

Supplementary Figure 11. Impact of the detection efficiency on *in-silico* contact maps.

Hi-C, SPRITE and GAM *in-silico* contact maps are shown for three different efficiencies (0.01, 0.1 and 1.0) for fixed N=1000 *in-silico* cells, in the *Sox9* locus case study. Low efficiencies can strongly disrupt the quality of the maps (see **Figure 5f**)

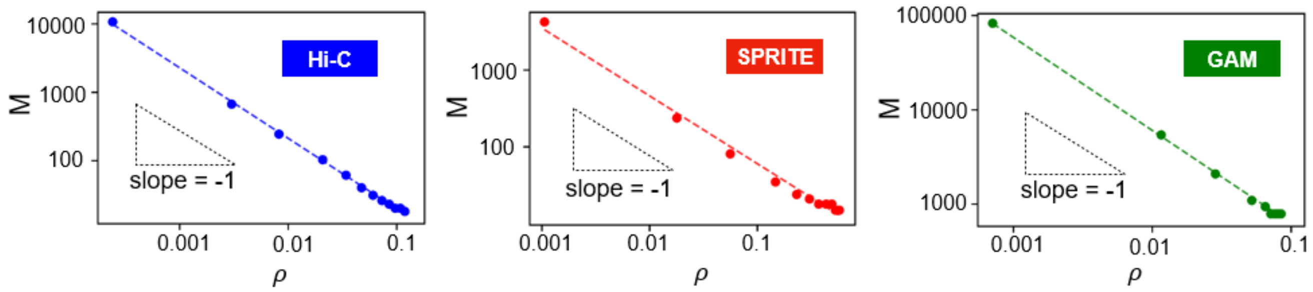

Supplementary Figure 12. The estimated value of M is consistent with the Central-Limit-Theorem

In the *Sox9* locus case study, the values of M (see **Main Text**, **Figure 5**) at different efficiencies are plotted against ρ, the squared signal-to-noise ratio averaged over all the entries of a single-cell contact map (**Methods**). This is done for Hi-C (left), SPRITE (middle) and GAM (right). In all three plots (in log-log scale) the trend of M vs ρ is well fitted by a linear relationship with slope -1 (dashed lines) as expected by arguments based on the Central Limit Theorem (**Methods**).

Sox9 locus, mESC, model from GAM data

a)

	Hi-C		
Hi-C	0.75	0.64	0.44
	r	rs	HiCRep

	SPRITE		
SPRITE	0.82	0.74	0.25
	r	rs	HiCRep

	GAM		
GAM	0.79	0.76	0.18
	r	rs	HiCRep

c)

bulk	Distance		
Hi-C	-0.51	-0.75	-0.44
SPRITE	-0.59	-0.83	-0.45
GAM	-0.95	-0.98	-0.82
	r	rs	HiCRep

b)

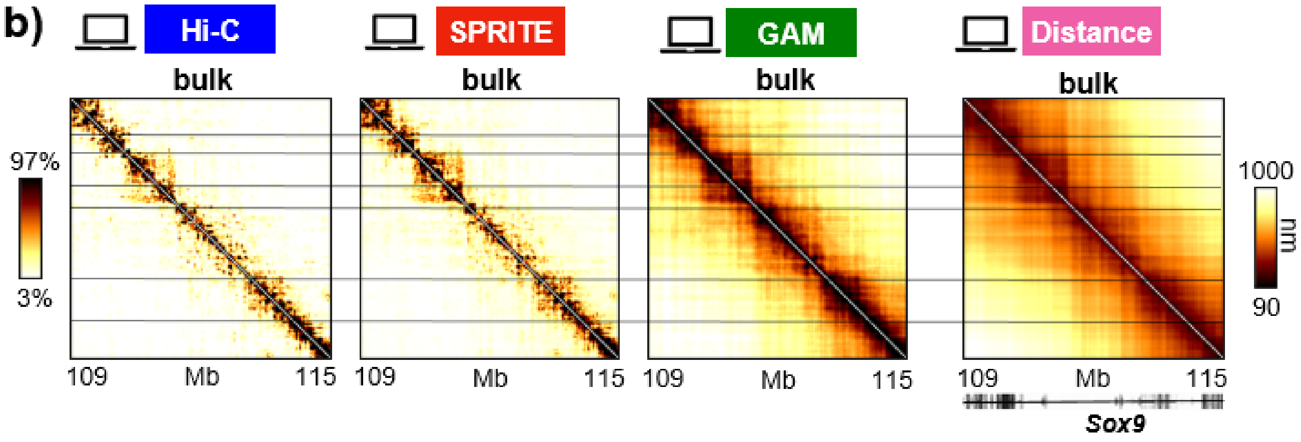

Supplementary Figure 13. *In-silico* contact and distance maps for the *Sox9* model derived from GAM data.

a) We considered 3D structures for the mESC *Sox9* locus derived[48] from GAM data[9] (**Methods**). The corresponding Hi-C, SPRITE and GAM *in-silico* contact maps are compared to the experimental data (the same used in **Figure 1**, see **Methods**) and their Pearson, Spearman and HiCRep correlations are reported.

b) The *in-silico* bulk contact maps are compatible with the average distance pattern obtained from the ensemble of GAM-derived 3D conformations. Horizontal lines are drawn to highlight the patterns detected across the contact and the distance maps. For the contact maps, color scale indicates the percentiles.

c) Pearson, Spearman and HiCRep correlations are reported between each bulk contact map and the average distance map.

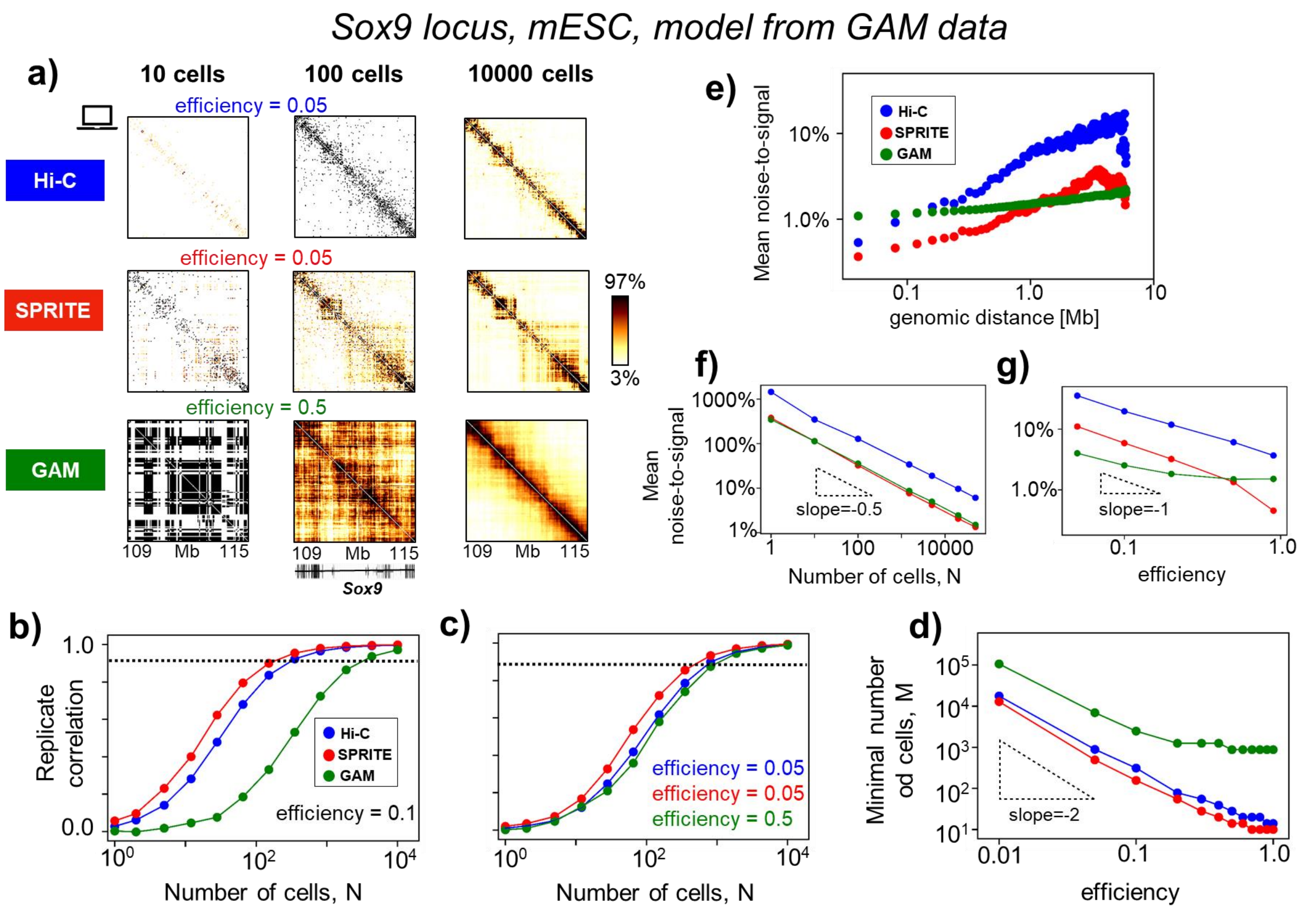

Supplementary Figure 14. Replicates reproducibility and noise level in the *Sox9* locus simulated from GAM data

a) The *in-silico* Hi-C, SPRITE and GAM contact maps depend on the number of cells used in the experiment. Efficiencies analogous to those found in real experiments are employed (**Figure 5b**). Color scale indicates the percentiles of each map.

b) The Pearson correlation is shown between replicates as function of N at a given efficiency (0.1). The dashed line is the threshold correlation value r_t=0.9 (**Main Text** and **Methods**).

c) Analogous results as in panel b) are found for efficiencies similar to those of real experiments (see panel a)).

d) The minimal number of cells, M, required for replicate reproducibility is shown against the efficiency for Hi-C, SPRITE and GAM. For small efficiencies, M varies approximately as an inverse squared power law as function of the efficiency, as expected from the Central Limit Theorem (**Methods**).

e) For fixed number of cells and efficiency (respectively N=50000 and efficiency 0.5), the average noise-to-signal ratio $\langle\sigma/\mu\rangle$ is reported against the genomic distance. The noise-to-signal ratio rises significantly above 1Mb for Hi-C and SPRITE.

f) For fixed genomic distance and efficiency (respectively 1Mb and efficiency 0.5), the noise-to-signal ratio decreases with the number of cells as an inverse square root, as expected from the Central

Limit Theorem.

g) For fixed genomic distance and number of cells (respectively for 1Mb and N=50000), the noise-to-signal ratio decreases approximately as an inverse power law when the efficiency increases, for all three technologies. All the above findings are consistent with those found for the *Sox9* locus simulated from Hi-C data (**Figures 5,6**) and all the other considered loci in murine and human cells.

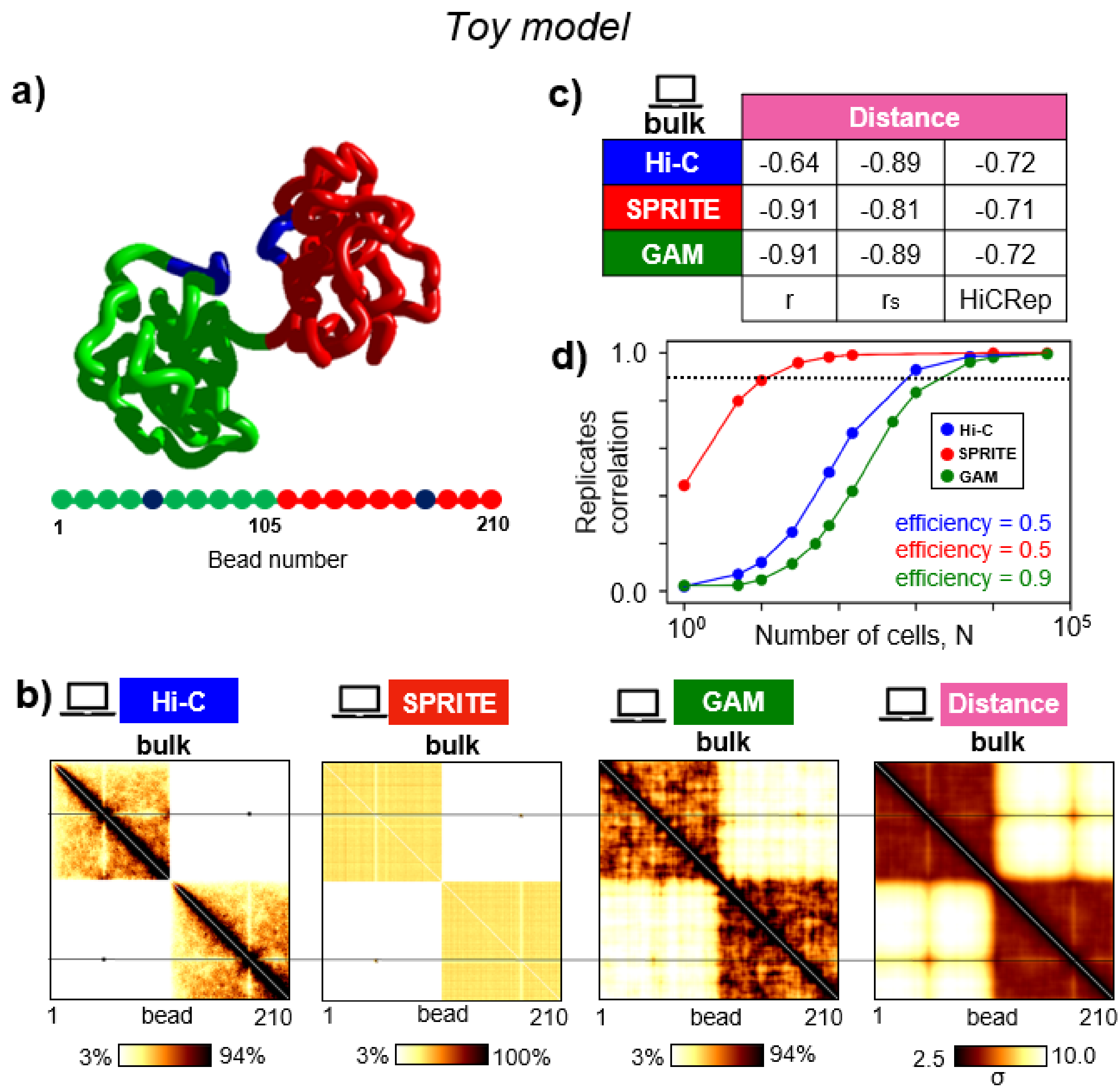

Supplementary Figure 15. *In-silico* maps from 3D conformations of a toy polymer model.

a) We considered a simple block copolymer model made of 210 beads where same colored regions attract each other (see **Methods**). The example of a 3D structure is shown.

b) *In-silico* Hi-C, SPRITE and GAM bulk contact maps all yield contact patterns compatible with the average distance pattern derived from our ensemble of conformations. The horizontal lines are a guide to the eye. Color scale for the contact maps indicates the percentiles. For the distance map, color scale is given in the units of σ, the diameter of a polymer bead (**Methods**).

c) The Pearson, Spearman and HiCRep correlations between each bulk contact and the average distance map are reported.

d) Replicate Pearson correlations are plotted v.s. the number of cells N, for efficiencies equal to 0.5 for Hi-C and SPRITE, 0.9 for GAM. All the above results are overall comparable to those obtained from the models of all the other loci analysed (**Figures 3,5; Supplementary Figures 1,2,4,8-10**).

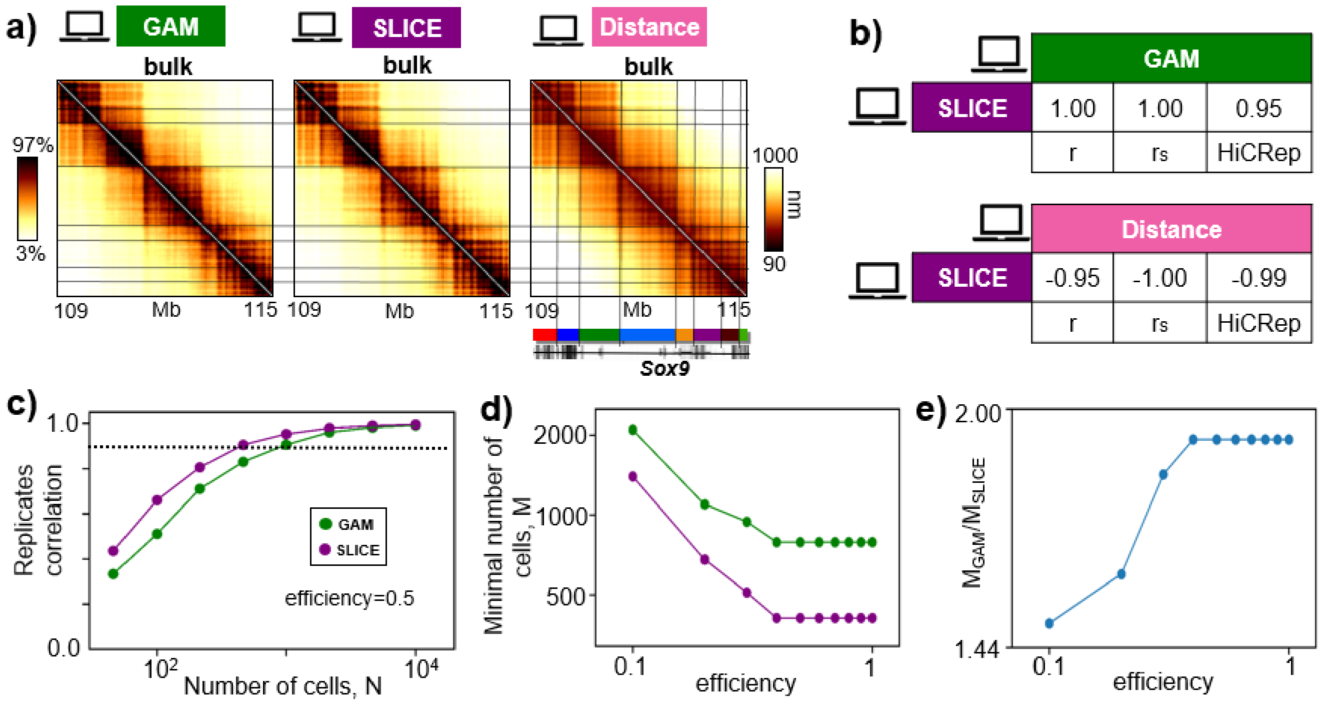

Supplementary Figure 16. The SLICE analysis tool for GAM is faithful to the benchmark distance map.

a) For the *Sox9* locus case study, the *in-silico* bulk GAM map and the corresponding SLICE map (**Main Text** and **Methods**) return consistent patterns. Color scale indicates the percentiles of the maps. In particular, the SLICE single-cell interaction probability map is also faithful to the average distance pattern. Horizontal lines highlight that GAM and SLICE both capture the domain structure of the distance map, corresponding to the TADs[19] shown in the color bar at the bottom.

b) Pearson, Spearman and HiCRep correlations between SLICE and GAM bulk maps (top) and between SLICE and the average distance maps (bottom).

c) The Pearson correlation between replicate contact maps is shown as a function of the number of cells, N, for GAM and SLICE at efficiency 0.5 (**Methods**).

d) The minimal number of cells, M, to have reproducible replicates at different efficiencies for SLICE and GAM (as in **Figure 5f**).

e) The ratio between M for GAM and for SLICE v.s. the efficiency. For efficiencies close to the experimental ones, say 0.5 or above[9], the value of M for SLICE is approximately a factor two lower than for GAM.
