# Supplementary material for: Comparison of the Hi-C, GAM and SPRITE methods using polymer models of chromatin
Source: Nat Methods. 2021 May 7;18(5):482–90. doi: 10.1038/s41592-021-01135-1 (PMC8416658; doi:10.1038/s41592-021-01135-1)
Supplement: Supplementary file 2 — Reporting Summary [file 41592_2021_1135_MOESM2_ESM.pdf]

nature research

Corresponding author(s): Mario Nicodemi
Last updated by author(s): Mar 1, 2021

Reporting Summary

Nature Research wishes to improve the reproducibility of the work that we publish. This form provides structure for consistency and transparency in reporting. For further information on Nature Research policies, see our Editorial Policies and the Editorial Policy Checklist.

Statistics

For all statistical analyses, confirm that the following items are present in the figure legend, table legend, main text, or Methods section.

n/a	Confirmed	
☐	☒	The exact sample size (n) for each experimental group/condition, given as a discrete number and unit of measurement
☐	☒	A statement on whether measurements were taken from distinct samples or whether the same sample was measured repeatedly
☐	☒	The statistical test(s) used AND whether they are one- or two-sided<br>*Only common tests should be described solely by name; describe more complex techniques in the Methods section.*
☒	☐	A description of all covariates tested
☐	☒	A description of any assumptions or corrections, such as tests of normality and adjustment for multiple comparisons
☐	☒	A full description of the statistical parameters including central tendency (e.g. means) or other basic estimates (e.g. regression coefficient) AND variation (e.g. standard deviation) or associated estimates of uncertainty (e.g. confidence intervals)
☐	☒	For null hypothesis testing, the test statistic (e.g. F, t, r) with confidence intervals, effect sizes, degrees of freedom and P value noted<br>*Give P values as exact values whenever suitable.*
☒	☐	For Bayesian analysis, information on the choice of priors and Markov chain Monte Carlo settings
☒	☐	For hierarchical and complex designs, identification of the appropriate level for tests and full reporting of outcomes
☐	☒	Estimates of effect sizes (e.g. Cohen's d, Pearson's r), indicating how they were calculated

Our web collection on statistics for biologists contains articles on many of the points above.

Software and code

Policy information about availability of computer code

Data collection	No software was used
Data analysis	The codes used in our work are based on standard, publicly available software packages, as detailed in the Methods section. Molecular Dynamics simulations use LAMMPS, version 30july2016. Analyses and plots were produced with the Anaconda package version 4.7.12. HiCRep correlations were computed with R, version 3.5.1. 3D structure visualisations were produced with POV-Ray, version 3.7. The algorithms for simulating Hi-C, SPRITE, GAM and SLICE in-silico also use standard routines, such as DBSCAN, and are described in full details in the Methods section and in the original papers. All the codes are available upon request.

For manuscripts utilizing custom algorithms or software that are central to the research but not yet described in published literature, software must be made available to editors and reviewers. We strongly encourage code deposition in a community repository (e.g. GitHub). See the Nature Research guidelines for submitting code & software for further information.

Data

Policy information about availability of data

All manuscripts must include a data availability statement. This statement should provide the following information, where applicable:
- Accession codes, unique identifiers, or web links for publicly available datasets
- A list of figures that have associated raw data
- A description of any restrictions on data availability

Published Hi-C, SPRITE,GAM and microscopy data used are available at the referenced papers in the manuscript. New GAM F123 data are available on the 4D Nucleome data portal under accession number 4DNFIFBSQ1EO.

Field-specific reporting

Please select the one below that is the best fit for your research. If you are not sure, read the appropriate sections before making your selection.

☒ Life sciences ☐ Behavioural & social sciences ☐ Ecological, evolutionary & environmental sciences

For a reference copy of the document with all sections, see nature.com/documents/nr-reporting-summary-flat.pdf

Life sciences study design

All studies must disclose on these points even when the disclosure is negative.

Sample size	The computational analyses were performed over previously published data of a 6Mb-long genomic locus at 40kb, a 7Mb-long genomic locus at 40kb, a 6Mb-long genomic locus at 10kb and a 2.5Mb-long genomic locus at 30kb. Then we also analysed a 210 beads long block-copolymer.
Data exclusions	No data were excluded for our analysis.
Replication	To verify the reproducibility of our findings, we applied our computational approach to four different genomic loci and on a block-copolymer and found analogous results.
Randomization	To test the significance of the HiCRep correlations (scc) between our model matrices and the experimental ones, we benchmarked against a control distribution of scc derived from 100 randomized matrices. We verified that the scc between our in-silico matrices and the corresponding experimental matrices are above the 90th percentile of the random control.<br>To test that our ensemble of model polymer structures is a bona-fide representation of real configurations of chromatin in nuclei, in the case of the human locus, we performed an RMSD analysis between available imaging data and our model structures and compared with the results from a random control, i.e. between the imaging data and self-avoiding-walk polymers. We found that the imaging-model RMSD distribution is statistically different than the control.
Blinding	This is not relevant for our study as we do not have clinical data from different patients.

Reporting for specific materials, systems and methods

We require information from authors about some types of materials, experimental systems and methods used in many studies. Here, indicate whether each material, system or method listed is relevant to your study. If you are not sure if a list item applies to your research, read the appropriate section before selecting a response.

Materials & experimental systems

n/a	Involved in the study	
☒	☐	Antibodies
☐	☒	Eukaryotic cell lines
☒	☐	Palaeontology and archaeology
☒	☐	Animals and other organisms
☒	☐	Human research participants
☒	☐	Clinical data
☒	☐	Dual use research of concern

Methods

n/a	Involved in the study	
☒	☐	ChIP-seq
☒	☐	Flow cytometry
☒	☐	MRI-based neuroimaging

Eukaryotic cell lines

Policy information about cell lines

Cell line source(s)	Mouse F123 ESCs (a male, hybrid cell line, derived from S129/Jae and Cast) (Gribnau et al., 2003) were kindly provided by Bing Ren, University of California San Diego, San Diego, CA, USA
Authentication	Whole genome sequencing confirmed positions of SNPs, immunofluorescence for cell stage markers confirmed pluripotent state of mESC line
Mycoplasma contamination	The used cell line was tested negative for mycoplasma
Commonly misidentified lines (See ICLAC register)	No commonly misidentified cells lines were used in this study
